# *ARID1A*‐deficient cells require HDAC6 for progression of endometrial carcinoma

**DOI:** 10.1002/1878-0261.13193

**Published:** 2022-03-02

**Authors:** Cristina Megino‐Luque, Pol Sisó, Natalia Mota‐Martorell, Raúl Navaridas, Inés de la Rosa, Izaskun Urdanibia, Manel Albertí‐Valls, Maria Santacana, Miquel Pinyol, Núria Bonifaci, Anna Macià, David Llobet‐Navas, Sònia Gatius, Xavier Matias‐Guiu, Núria Eritja

**Affiliations:** ^1^ Oncologic Pathology Group Department of Basic Medical Sciences Biomedical Research Institute of Lleida (IRBLleida) University of Lleida Spain; ^2^ Centro de Investigación Biomédica en Red de Cáncer (CIBERONC) Madrid Spain; ^3^ Oncologic Pathology Group Department of Medicine Biomedical Research Institute of Lleida (IRBLleida) University of Lleida Spain; ^4^ Metabolic Physiopathology Group Department of Experimental Medicine Biomedical Research Institute of Lleida (IRBLleida) University of Lleida Spain; ^5^ 16296 Scientific and Technical Service of Immunohistochemistry Biomedical Research Institute of Lleida (IRBLleida) Hospital Universitari Arnau de Vilanova Lleida Spain; ^6^ 16296 Department of Pathology Hospital Universitari Arnau de Vilanova Lleida Spain; ^7^ Molecular Mechanisms and Experimental Therapy in Oncology‐Oncobell Program Bellvitge Biomedical Research Institute (IDIBELL) Barcelona Spain; ^8^ Oncologic Pathology Group Department of Experimental Medicine Biomedical Research Institute of Lleida (IRBLleida) University of Lleida Spain; ^9^ Department of Pathology Hospital Universitari de Bellvitge IDIBELL University of Barcelona Spain

**Keywords:** ACY1215, ARID1A, endometrial cancer, HDAC6

## Abstract

AT‐rich interactive domain‐containing protein 1A (ARID1A) loss‐of‐function mutation accompanied by a loss of ARID1A protein expression is frequently observed in endometrial carcinomas. However, the molecular mechanisms linking these genetic changes to the altered pathways regulating tumour initiation, maintenance and/or progression remain poorly understood. Thus, the main aim of this study was to analyse the role of ARID1A loss of function in endometrial tumorigenesis. Here, using different endometrial *in vitro* and *in vivo* models, such as tumoral cell lines, 3D primary cultures and metastatic or genetically modified mouse models, we show that altered expression of *ARID1A* is not enough to initiate endometrial tumorigenesis. However, in an established endometrial cancer context, ARID1A loss of function accelerates tumoral progression and metastasis through the disruption of the G2/M cell cycle checkpoint and ATM/ATR‐mediated DNA damage checkpoints, increases epithelial cell proliferation rates and induces epithelial mesenchymal transition through the activation of histone deacetylase 6 (HDAC6). Next, we demonstrated that the inhibition of HDAC6 function, using the HDAC6‐specific inhibitor ACY1215 or by transfection with *HDAC6* short hairpin RNA (shRNA), can reverse the migratory and invasive phenotype of *ARID1A‐*knockdown cells. Further, we also show that inhibition of HDAC6 activity causes an apoptotic vulnerability to etoposide treatments in *ARID1A‐*deficient cells. In summary, the findings exposed in this work indicate that the inhibition of HDAC6 activity is a potential therapeutic strategy for patients suffering from ARID1A‐mutant endometrial cancer diagnosed in advanced stages.

## Graphical abstract

Although endometrial carcinoma is considered to have a good prognosis, more than 20% of women die from their disease because they are diagnosed at advanced stages. Here, we show that deficient expression of ARID1A confers increased aggressiveness and metastatic capacity to endometrial cancer cells, while increasing HDAC6 levels. The use of HDAC6 inhibitors could represent a therapeutic strategy for patients diagnosed with advanced stages of ARID1A‐mutated tumours.

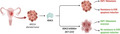

**Abbreviations**
ARID1A: AT‐rich interactive domain‐containing protein 1A
ATM: ataxia‐telangiectasia mutated
ATR: ataxia telangiectasia and Rad3‐related protein
BrdU: 5‐bromo‐2′‐deoxyuridine
CDC25C: cell division cycle 25C
CDK: cyclin‐dependent kinase
CHK: checkpoint kinase
CKI: CDK inhibitors
Cre:ERT: tamoxifen‐inducible cre estrogen receptor recombinase
DNA: deoxyribonucleic acid
DNA‐PKcs: DNA‐dependent protein kinase, catalytic subunit,
DSB: double‐strand breaks
EC: endometrial cancer
EEC: endometrioid endometrial cancer
EGFP: enhanced green fluorescent protein
EMT: epithelial–mesenchymal transition
ERK: extracellular signal‐regulated kinases
EV: empty vector
GSEA: gene set enrichment analysis
HDAC6: histone deacetylase 6
HES: human uterine epithelial cells
HR: homologous recombination
IK: Ishikawa
JNK: jun amino‐terminal kinases
MAPK: mitogen‐activated protein kinase
MMP: matrix metalloproteinase
MNK: mitogen‐activated protein kinase interacting protein kinases
NEEC: non‐endometrioid endometrial cancer
NHEJ: non‐homologous end‐joining
OCCC: ovarian clear cell carcinoma
PARP: poly ADP ribose polymerase
RNA: ribonucleic acid
SAPK: stress‐activated protein kinases
sgRNA: single guide RNA
shRNA: short hairpin RNA
SNAIL1: snail family transcriptional repressor 1
SPF: specific pathogen‐free
SWI/SNF: switch/Sucrose non‐fermentable
XRCC4: x‐ray repair cross complementing 4
ZEB: zinc finger E‐box‐binding homeobox 1
γH2AX: phosphorylation of the histone H2AX

## 1 Introduction

Endometrial cancer (EC) is the most frequent infiltrating tumour of the female genital tract and the sixth most common cancer in women worldwide [1]. Furthermore, it is one of the few cancers whose incidence and mortality have increased, probably related to the increasing life span and obesity rates [2]. Although most endometrial cancers are diagnosed at early treatable stages, approximately 20% of the patients will present late diagnosis at advanced stages and 8% of them will have distant metastasis, altogether resulting in poor prognosis [3]. Conventionally, ECs have been broadly classified into two groups based on clinical, pathological and molecular features: Type I or endometroid EC (EEC) subtype, composed mostly by endometrioid histology, representing approximately 85% of all EC and usually associated with favourable prognosis and type II or non‐endometrioid EC (NEEC) subtype, accounting for ~ 15% of EC, with a poor clinical outcome [4, 5]. Although the dualistic classification is broadly used, it is not entirely robust since some endometrial cancers present characteristics of both types. In 2013, a new molecular classification of EC profiling shed light on four different EC genomic subtypes, detecting high frequencies of somatic mutations in well‐known cancer genes such as *ARID1A* (33%) [6].

The SWI/SNF chromatin remodelling complex modulates gene transcription, by remodelling nucleosomes in an ATP dependent manner and is involved in diverse cellular processes such as tissue differentiation, proliferation and DNA repair [7]. The SWI/SNF complex is mutated in > 20% of all human cancers, and the *ARID1A* (At‐rich interactive domain, also known as BAF250A) subunit is especially prone to somatic mutations in gynaecologic cancers, although its role in endometrial tumorigenesis is not fully understood [8, 9, 10, 11]. On one hand, some studies have shown that *ARID1A* gene mutations are present in 40% of low‐grade ECs and ARID1A protein expression is lost in ~ 25% [8, 12, 13, 14]. Moreover, loss of ARID1A expression has been observed in focal areas of atypical endometrial hyperplasia [15, 16]. All these studies indicate that loss of ARID1A is associated with oncogenic cell transformation, which suggests a tumour suppressor role for ARID1A in neoplasms originating from the endometrium. On the other hand, several authors have documented that loss of ARID1A expression in endometrial biopsy or curettage is associated with a significantly increased‐risk and higher FIGO stages of ECs in subsequent hysterectomies [17]. A study performing whole‐exome sequencing of 98 tumour biopsies from primary tumours and their paired abdominopelvic metastases demonstrated that *ARID1A* was frequently mutated in the branches of EC phylogenesis [18], suggesting that ARID1A is involved in EC tumour progression rather than its initiation. Thus, understanding the role of ARID1A in initiation/progression of ECs may facilitate the development of tailored treatment and improved patient management.

ARID1A has been present in many crucial processes in carcinogenesis, including cell proliferation, epithelial‐mesenchymal transition (EMT), migration activity and invasion capability [19]. Moreover, ARID1A has been linked to classical mechanisms of tumour suppression, since ARID1A roles include regulation of cell cycle, DNA damage checkpoint and P53 targets [20]. Some authors have suggested that ARID1A has an important role in the DNA damage response by maintaining genome integrity, by sustaining checkpoint activation and by facilitating and/or accelerating effective DNA double‐strand (DSB) end resection [21]. Thus, loss of ARID1A may play an important role in DNA repair efficacy in the acquisition of the tumoral phenotypes. Nevertheless, which molecular pathways are employed by ARID1A‐deficient tumours to survive and maintain DNA integrity in response to endogenous or exogenous events that result in DNA damage remains to be determined.

The exposed conflicting data of ARID1A role in endometrial carcinogenesis and the fact that ARID1A directly suppresses HDCA6 provide evidence that HDAC6 may be a promising therapeutic target in ARID1A‐mutated cancers. We sought to delineate the mechanistic roles of ARID1A in endometrial tumorigenesis to translate these findings into improved strategies for the treatment of patients with EC.

## 2 Materials and methods

### 2.1 Reagents and antibodies for western blot

The following reagents were used: Etoposide (Sigma‐Aldrich, St. Louis, MO, USA, E1383); ACY‐1215 (MedChemExpress, Monmouth Junction, NJ , USA, HY‐16026); anti‐GAPDH (Abcam, Cambridge, MA, USA, 8245); anti‐ARID1A (Cell Signaling, Danvers, MA, USA, 12354); anti‐p16 (Santa Cruz, Dallas, TX, USA, sc‐1661); anti‐p21 (Santa Cruz, sc‐817); anti‐Cyclin D1 (Santa Cruz, sc‐20044); anti‐Cyclin E (Santa Cruz, sc‐247); anti‐cyclin A2 (Santa Cruz, sc‐271682); anti‐cyclin B (Santa Cruz, sc‐166210); anti‐CDK2 (Santa Cruz, sc‐748); anti‐CDK 4/6 (Abcam, 3112); anti‐CDK1 (Santa Cruz, sc‐8395); anti‐phospho‐CDK1 (Tyr15) (Santa Cruz, sc‐136014); anti‐phospho‐chk2 (Thr68) (Cell signaling, 2197); anti‐chk1 (Ser 345) (Cell Signaling, 2348); anti‐p‐53 (Ser15) (Cell Signaling, 9284); anti‐Ac‐p53 (Lys 373‐382) (Sigma‐Aldrich, 06‐916); anti‐p53 (Santa Cruz, sc‐126); anti‐CDC25 (Santa Cruz, sc‐13138); anti‐cleaved Caspase‐3 (Cell Signaling, 9661); anti‐cleaved PARP (Cell Signaling, 5625); anti‐E‐cadherin (BD Biosciences, San Jose, CA, USA, 610181); anti‐β‐catenin (BD Biosciences, 610153); anti‐Vimentin (BD Bioscience, 550513); anti‐MMP2 (Santa Cruz, sc‐13594); anti‐SNAIL (Cell Signaling, 3879); anti‐ZEB (RF); anti‐phosph‐ERK 1/2 (Thr202/Tyr204) (Biolegend, San Diego, CA, USA, 22.675505); anti‐pan‐ERK1/2 (BD Biosciences, 610623); anti‐phospho‐SAPK/JNK (Thr183/185) (Cell Signaling, 9251); anti‐SAP/JNK (Cell Signaling, 9258); anti‐phopho‐p38 α/β (Thr180/Tyr185) (Cell Signaling, 4511); anti‐p38 α/β (Santa Cruz, sc‐7972); anti‐c‐Fos (Cell Signalling, 5348); anti‐phospho‐MNK1 (Thr197/202) (Cell Signaling, 2111); anti‐HDAC6 (CellSignaling, 7612); anti‐Ku70 (Santa Cruz, sc‐17789) and anti‐XRCC4 (Santa Cruz, sc‐5282); anti‐p‐ATM (S1981) (Abcam, ab208775); anti‐ATM (Santa Cruz, sc‐135663); anti‐Phospho‐ATR (Ser428) (CellSignalling, 2853); ATR (Santa Cruz, sc‐515173); anti chk2 (Santa Cruz, sc‐5278); anti chk1 (Santa Cruz, sc‐8408); anti PARP (Santa Cruz, sc‐8007).

### 2.2 Cell culture

Human uterine epithelial cells (HES) are characterized as a spontaneously immortalized cell line derived from benign proliferating endometrium isolated via hysterectomy and were obtained from Dr Doug Kniss at Ohio State University [22]. Human endometrioid EC cell lines HEC‐1a, and AN3CA, were purchased from American Type Culture Collection (2008; ATCC‐authentication by isoenzymes analysis). Ishikawa 3‐H‐12 (IK) were purchased from Sigma‐Aldrich, 99040201 as well as MFE‐296 cell line (Sigma‐Aldrich, 98031101). Cells were grown in Dulbecco’s modified Eagle’s medium (DMEM; Sigma‐Aldrich, 12007559) supplemented with 10% fetal bovine serum (FBS; Invitrogen, Waltman, MA, USA, 10270106), 1 mmol·L^−1^ HEPES (Sigma‐Aldrich, H3375), 1 mmol·L^−1^ sodium pyruvate (Sigma‐Aldrich, P2256), 2 mmol·L^−1^ L‐glutamine (Sigma‐Aldrich, C59202), 1% penicillin/streptomycin (Sigma‐Aldrich, P4333) at 37 °C with saturating humidity and 5% CO_2_.

To generate HEC‐1A, Ishikawa 3‐H‐12 (IK) and MFE‐296 sgRNA *ARID1A‐*deficient cell lines, cells were infected with the lentiviral plasmid encoding Cas9 and the sgRNA against *ARID1A*. Cells infected with viruses encoding the puromycin resistance gene were selected in 2 μg·mL^−1^ puromycin.

### 2.3 3D spheroids cultures of EC cell lines

Human endometrial epithelial cell lines in cultures were grown as described previously with a minor modification [23]. Briefly, cells were washed with Hanks Balanced Salt Solution (HBSS) (Thermo Fisher Scientific, Waltman, MA, USA, 11520476) and incubated with trypsin‐EDTA solution (Sigma‐Aldrich, T4049) for 3 min at 37 °C. Trypsin activity was stopped by adding DMEM containing 10% FBS. Cells were centrifuged at 18 ***g*** for 3 min and diluted in DMEM‐F12 medium (Sigma‐Aldrich, 11580376) containing 3% Matrigel (BD Biosciences, 354234) and 2% dextran‐coated charcoal stripped serum (HyClone Laboratories, Logan, UT, USA, 11571821), obtaining 1 × 10^4^ cell·mL^−1^. Cells were cultured for 4–7 days in an incubator at 37 °C with saturating humidity and 5% CO_2_. For immunofluorescence, cells were seeded in a volume of 40mL per well in 96‐well black plates with a microclear bottom (Greiner Bio‐One, Madrid, Spain, 655090).

### 2.4 Viral production, infection and *in vitro* transfection conditions

To produce plasmid‐based sgRNA, oligonucleotides were cloned into the lentiviral lentiCRISPRv2 vector using BsmBI restriction sites. sgRNA targets sequence were: ARID1A.1 5′‐ CACCGATGTTGTTGGTGGAAGACGG; ARID1A.2 5′‐ CACCGGCTTTCTTCAGCTCCGAGGG; ARID1A.3 5′‐ CACCGTATGGCCAATATGCCACCTC. Target sequences were functional against both human *ARID1A* and mice *Arid1a* genes. To target HDAC6, plasmids carrying shRNA HDAC6 were employed (abm, Richmond, Canada, 2313409).

Production of viral particles was achieved by transfecting HEK‐293 packaging cells with linear PEI (40 µm) in combination with lentiviral plasmids and helper plasmids (psPAX2 packaging and pMD2G envelope) at 1 : 1 : 1 ratio, respectively.

Four hours after transfection, packaging cells were cultured with DMEM supplemented with 10% FBS, 1 mmol·L^−1^ HEPES (Sigma‐Aldrich), 1 mmol·L^−1^ sodium pyruvate (Sigma‐Aldrich), 2 mmol·L^−1^ L‐glutamine (Sigma‐Aldrich) and 1% of penicillin/streptomycin (Sigma‐Aldrich) for 3–4 days; afterwards, the medium containing the viral particles was collected, centrifuged for 10 min at 200 ***g*** and filtered thought a 0.45 µm filter (Millipore, Temecula, CA, USA) and concentrated using Vivaspin concentrators (Sartorius Stedim Biotech GmbH, Gottingen, Germany). The concentrated medium containing lentiviral particles was added to the medium of the pre‐plated cells. Cells were incubated for 24 h. After this period, the medium was replaced with fresh medium and cells were grown regularly to allow phenotypic expression.

### 2.5 Isolation and three‐dimensional glandular cultures from mice epithelial endometrial cells

The isolation and 3D culture of endometrial epithelial cells were performed using a previously described method with minor modifications [23].

When required, *Arid1a* deletion in endometrial cells isolated from *Cre:ER* ^+/−^; *Arid1a^fl/fl^* mice, were induced by addition of 0.5 µg·mL^−1^ of tamoxifen in cell culture medium.

### 2.6 Genetically modified mouse models

The *in vivo* studies complied with Law 5/1995 and Act 214/1997 of the Regional Government (Generalitat de Catalunya) and EU Directive EEC 63/2010 and were approved by the Ethics Committee on Animal Experiments of the University of Lleida and the Ethics Commission in Animal Experimentation of the Generalitat de Catalunya. *Cre‐ER^T^* (B6). Cg‐Tg(CAG‐Cre/Esr1*5Amc/J) mice were obtained from the Jackson Laboratory (Bar Harbor, ME, USA). *Arid1a* ^f/f^ mice were a kind gift from Dr. I. Lei. Mice bearing floxed *Arid1a* allele in which exon 9 of *Arid1a* gene is flanked by loxP sites have been described before [24]. *Arid1a^f/f^* mice were backcrossed for five generations with C57BL/6 mice before being crossed with *Cre‐ER^T^* transgenic strains to generate epithelial cell‐specific deletion of Arid1a. Mice were genotyped by earmarking, and DNA was isolated from tail tissue in proteinase K lysis buffer. PCR was carried out with GoTap polymerase (Promega, Madison, WI, USA) using different pairs of primers for each gene. *Cre‐ER^+/−^* forward primer 5′‐ACGAACCTGGTCGAAATCGTGCG‐3′ and reverse primer 5′‐CGGTCGATGCAACGAGTGATGAG‐3′; *Arid1a^f/f^* forward primer 5′‐GGCTCTGCCATAAAGCGATCC‐3′ and reverse primer 5′‐CTCACAAATCTAACCGAGGCCAC‐3′.

### 2.7 Subcutaneous tumor xenografts and retro‐orbital metastasis model injections

Immunodeficient 12‐week‐old female (NSG), h/h mice (weight 20–25 g) were maintained in specific pathogen‐free (SPF) conditions and manipulated in accordance with institutional guidelines approved by the Biomedical Research Institute of Lleida (IRB Lleida) regional committee for animal care. Animals were subcutaneously injected with Ishikawa cells (1.5 × 10^6^) resuspended in 100µl PBS + Matrigel^®^ (1 : 1). Tumors were allowed grow for 14 days Tumors were measured 3 times per week with calipers. Tumor size was calculated as (*D* × *d*2)/2 D mm^3^ [25]. Etoposide was administered intraperitoneally 3 times per week (36 µm).

### 2.8 Retro‐orbital metastasis model

Immunodeficient 12‐week‐old female (NSG), h/h mice (weight 20–25 g) were maintained in specific pathogen‐free (SPF) conditions and manipulated in accordance with institutional guidelines approved by the Biomedical Research Institute of Lleida (IRB Lleida) regional committee for animal care. Retro‐orbital metastasis model: 50 × 10^4^ MFE‐296 cells expressing EGFP‐luciferase were injected retro‐orbitally into the sinus of immunodeficient NSG females. Retro‐orbital injections were conducted under 2% isoflurane/air anaesthesia. A successful retro‐orbital injection was confirmed on day 0 by images showing systemic bioluminescence distributed throughout the animals; 6–10 females per group with evidence of a satisfactory injection continued the experiment. Ten days after the injection, we monitored tumour lesions by Photon Imager (Biospace, Urbandale, IA, USA) coupled with live imaging software M3 Vision Viewer. For bioluminescence tumour imaging; luciferin (Caliper Life Science, Houston, TX, USA, 119222) was used as the substrate for the luciferase expressing tumour cells and was injected intraperitoneally at 150 mg·kg^−1^ in PBS. The extended method has been described before [25].

### 2.9 Immunohistochemical study

After sacrifice, mice uteri were excised, flushed with PBS, fixed in 10% neutral‐buffered formalin and embedded in paraffin. Mice uterus blocks were sectioned for a thickness of 3 μm, were dried for 1 h at 65 °C before being pre‐treated for deparaffinization, rehydration and epitope retrieval in the pre‐treatment Module, PT‐LINK (Agilent Technologies‐DAKO, Santa Clara, CA, USA) at 95 °C for 20 min in 50 × Tris/EDTA buffer, pH 9. Before staining the sections, endogenous peroxidase was blocked. The antibodies used were anti‐ARID1A (1 : 500 dilution, Abcam, ab182561) and Ki‐67 (1 : 100 dilution, Abcam, ab16667). After incubation, the reaction was visualized with the EnVisionTM FLEX+ rabbit (Linker) Detection Kit (Agilent Technologies‐DAKO) for ARID1A and the secondary antibody polyclonal goat anti‐rabbit IgG/Biotin (1 : 200 dilution, Jackson Immunoresearch, 111‐065‐144) plus Streptavidin/HRP (1 : 400 dilution, Agilent Technologies‐DAKO, P0397) for Ki67, using diaminobenzidine chromogen as a substrate. Sections were counterstained with hematoxylin. Appropriate negative controls including no primary antibody were also tested.

Immunohistochemical results were evaluated by following uniform pre‐established criteria. Immunostaining was graded semi‐quantitatively by considering the percentage and intensity of the staining. A histological score was obtained from each sample and values ranged from 0 (no immunoreaction) to 300 (maximum immunoreactivity). The score was obtained by applying the following formula, Histoscore = 1 × (% light staining) + 2 × (% moderate staining) + 3 × (% strong staining). The histological score was also used for evaluation of cytosolic and nuclear staining intensity. To support the scoring of immunohistochemistry, a digital slide scanner [Nuclear Quant Module, Pannoramic 250 FLASH II 2.0 (3D HISTEC)] was used and the percentage of positive cells was determined.

### 2.10 Cumulative population doubling assay

For the measurement of cumulative population doublings (PDs), cells were plated at a density of 1 × 10^4^ cells per well in 6‐well plates. Cells were counted every 2 days for a period of 15 days. Next, a growth curve was drawn and the PD was calculated using the equation (*t*2 − *t*1)/3,32 × (log *n*2 − log *n*1).

### 2.11 Transwell assay

Cells were plated in the upper chamber of the Transwell (8 µm pore, Falcon, Glendale, AZ, USA) coated with Matrigel in serum‐free medium at a density of 1 × 10^4^ per well. FBS 10% was used as a chemoattractant. After 48 h, cells were fixed with paraformaldehyde 4% and stained with Hoechst (5 µg·mL^−1^). Finally, cells were pictured with an epifluorescence microscope (Leica, Wetzlar, Germany), before and after a sterile cotton wipe. Results were analyzed to obtain the percentage of invasive cells using the software image j (NIH, Bethesda, MD, USA).

### 2.12 Wound healing assay

For migration, 6 × 10^4^ cells were plated in triplicates 24 well plates. When the cells reached ~ 70% confluence, a wound was scratched with a 200 µm pipette tip. Images were taken immediately after the scratch and 48 h later to calculate the percentage of closed wound using the software image j.

### 2.13 Total RNA extraction, reverse transcriptase‐PCR and quantitative real‐time

For RT‐qPCR, total RNA was extracted from the 3D or 2D cultures using the RNeasy Total RNA kit (Qiagen, Valencia, CA, USA) and cDNA was generated using the High‐Capacity cDNA Archive Kit (Applied Biosystems, Foster City, CA, USA). The obtained cDNA products were used as a template for subsequent RT‐qPCR assays using Taqman probes. The cDNA was amplified by heating to 95 °C for 15 s and 60 °C for 1 min using the ABI Prism 7900 Sequence Detection System (Applied Biosystems) and Promega GoTaq qPCR Master Mix. Relative mRNA expression levels were calculated using the 2^ΔΔ^ *^C^* ^t^ method and are presented as ratios to the housekeeping gene *GAPDH*. Taqman technology from Applied Biosystems was used for real‐time RT‐qPCR analyses. Probes used were: human *ARID1A*, Hs_00195664_m1; mouse *Arid1a*, Mm00473838_m1; human *HDAC6*, Hs_00997427_m1; mouse *Hdac6*, Mm00515945_m1; human *GAPDH*, Hs99999905_m1; mouse *Gapdh*, Mm99999915_g1.

### 2.14 Bromodeoxyuridine incorporation assay

For the determination of DNA and after the indicated treatments, 3D and 2D cultures were incubated with 3 ng·mL^−1^ of 5‐bromodeoxyuridine (BrdU, Sigma‐Aldrich), for 15 h for 3D cultures and 1 h for the 2D cultures, and then fixed with 4% paraformaldehyde for 20 min at room temperature. DNA denaturation was performed with 2 mol·L^−1^ HCl for 30 min. Afterwards, neutralization was done with 0.1 mol·L^−1^ Na2B4O7 (pH 8.5) for 2 min and then rinsed three times with PBS. Subsequently, block cells were placed in PBS solution containing 5% horse serum, 5% FBS, 0.2% glycine and 0.1% Triton X‐100 for 1 h. Further analysis was performed as previously described [23].

### 2.15 Confocal imaging and evaluation of Spheroid perimeter

Images of endometrial epithelial spheroids were captured and digitized by confocal microscope (Fluoview FV1000, Olympus, Tokyo, Japan). Epithelial perimeter analysis was processed by image analysis software (imagej version 1.46r), generating binary images of the spheroids as previously described [23].

### 2.16 Immunofluorescence assay

2D or 3D cultures were fixed with paraformaldehyde 4% for 15 min at room temperature and washed twice with PBS. Depending on the primary antibody, cells were permeabilized with 0.2% Triton X‐100 in PBS for 10 min or with 100% methanol for 5 min. Next, cultures were incubated overnight at 4 °C with the indicated diluted primary antibodies: anti‐phospho‐γ H2AX (Ser139) (1 : 200, methanol; Sigma‐Aldrich, 05‐636); anti‐phospho‐Histone H3 (Ser10) (1 : 200, methanol; BD Biosciences, 556433); anti‐cleaved caspase3 (1 : 200, Triton X‐100; Cell signaling, 9661); anti‐CDH1/E‐Cadherin (1 : 200, Triton X‐100; BD Biosciences, 610181); anti‐GOLGA2/GM130 (1 : 100, methanol; BD Biosciences, 610822); rhodamine‐conjugated phalloidin (1 : 500, Triton X‐100; Sigma‐Aldrich, P1951); anti‐β‐catenin (1 : 200, Triton X‐100; BD Biosciences, 610153); anti‐vimentin (1 : 200, methanol; BD bioscience, 550513); anti‐wide spectrum Cytokeratin (1 : 200, methanol; Abcam, ab9377); anti‐HDAC6 (1 : 200, Triton X‐100; Abcam, ab1440); anti‐α‐tubulin (1 : 500, Triton X‐100; Sigma‐Aldrich, T9026). Then, cells were washed twice with PBS and incubated with PBS containing 5μg·mL^−1^ of Hoechst 33342 and a 1 : 500 dilution of secondary anti‐mouse Alexa Fluor 546 (Invitrogen, A11005) and Alexa Fluor 488 (Invitrogen, A11029) or anti‐rabbit antibodies Alexa Fluor 594 (Invitrogen, R37119) and Alexa Fluor 488 (Invitrogen, A11034) for 4 h at room temperature. Immunofluorescence stains were visualized and analyzed using confocal microscopy (model FV1000; Olympus) with the 10×, 20× and the oil‐immersion 60× magnification objectives. Analysis of obtained images was performed with fluoview fv100 software (Olympus).

### 2.17 Western blotting

Western blotting analysis were performed as described previously [26]. Briefly, cells were washed with cold PBS and lysed with lysis buffer (2% SDS, 125 mmol·L^−1^ Tris‐HCl, pH 6.8). Equal amounts of proteins were subjected to SDS‐polyacrylamide gel electrophoresis and transferred to polyvinylidene difluoride membranes. Non‐specific binding was blocked by incubation with TBST (20 mm Tris‐HCl (pH 7.4), 150 mm NaCl and 0.1% Tween 20) plus 5% of non‐fat milk. Membranes were incubated with the primary antibodies overnight at 4 °C and for 1 h at room temperature with secondary horseradish peroxidase (1 : 10 000 in TBST). Signal was detected with SuperSignal West Femto Trial Kit (Thermo Scientific). Band analysis and densities shown in Fig. S7 were determined by using image lab 4.0.1 software (Bio‐Rad Laboratories, Richmond, CA, USA).

### 2.18 Analysis of gene expression endometrial carcinoma data sets

Gene expression data (RSEM) and their related clinical information of TCGA‐UCEC [27] (1) patients were downloaded from cbioportal [28, 29] and used to identify the samples in fifth quintile (20% High) and those in the first quintile (20% Low) according to ARID1A expression values. TCGA‐UCEC raw counts downloaded from Xena Browser [30] were log2 inversed and used to calculate differential expression analysis between high and low ARID1A expression samples using DESeq2 [31]. Then, transcripts were annotated to genes using the org.Hs.eg.db r package, keeping the transcript with the lowest *P*‐value per gene. Finally, genes were ranked according to the *P*‐values and the pre‐ranked GSEA [32] was implemented using default parameters and gene sets from MsigDB [33], in particular, KEGG, Ontology and Hallmark gene sets.

### 2.19 Detection of changes in cell‐cycle profiles

Changes in cell‐cycle profile after drug treatments were determined by propidium iodide staining and flow cytometry. Following treatment, approximately 1 × 10^6^ cells were fixed in 70% ethanol for at least 1 h on ice. The cells were then resuspended in 2 mL of cell‐cycle buffer (20 mg·mL^−1^ propidium iodide, in PBS containing 0.1% Triton X‐100 and 50 mg·mL^−1^ RNAase A) for 1 h at 37 °C. Propidium iodide fluorescence emission was measured using a FACSCanto II (BD Biosciences), and cell cycle distribution was analyzed with modfit lt software (Verity Software House, Topsham, ME, USA).

## 3 Results

### 3.1 Loss of ARID1A expression does not initiate endometrial malignant transformation

**Fig. 1:**
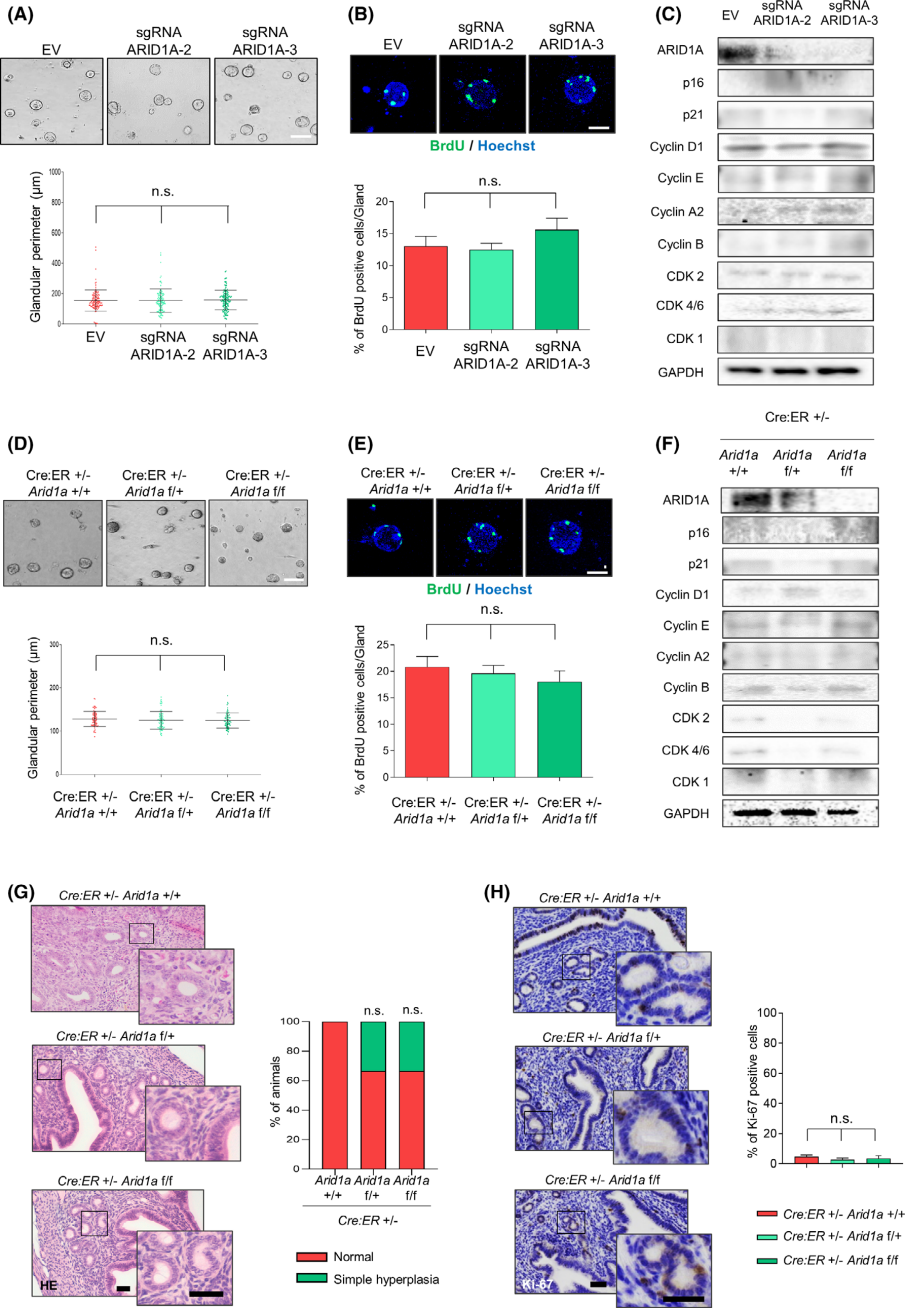
Loss of ARID1A expression does not initiate endometrial malignant transformation. (A) Representative phase contrast images and measurement of gland perimeter corresponding to 3D cultures of mice endometrial epithelial cells infected with lentiviruses carrying sgRNA against *Arid1a* (lentiCRISPRv2‐ARID1A.2 and lentiCRISPRv2‐ARID1A.3). Scale bars: 100 μm. Statistical analysis was performed using one‐way ANOVA analysis followed by the Tukey’s multiple comparison test. (B) Representative images and quantification of BrdU‐positive cells in 3D cultures of mice endometrial epithelial cells infected with lentiviruses carrying sgRNA against *Arid1a* (lentiCRISPRv2‐ARID1A.2 and lentiCRISPRv2‐ARID1A.3). Nuclei were evidenced by Hoechst staining. Scale bars: 25 μm. Statistical analysis was performed using one‐way ANOVA analysis followed by the Tukey’s multiple comparison test. (C) Representative images of western blot analysis of 3D cultures infected with lentiviruses carrying sgRNA against *Arid1a* (lentiCRISPRv2‐ARID1A.2 and lentiCRISPRv2‐ARID1A.3) showing ARID1A, p16, p21, Cyclin D1, Cyclin E, Cyclin A2, Cyclin B, CDK2, CDK4/6 and CDK1 protein expression. GAPDH was used as a loading control. Statistical analysis was performed using one‐way ANOVA analysis followed by the Tukey’s multiple comparison test. (D) Representative phase contrast images and measurement of gland perimeter corresponding to 3D cultures from *Cre:ER* ^T^ *; Arid1a^+/+^*, *Cre:ER* ^T^ *; Arid1a^f/+^* or *Cre:ER* ^T^ *; Arid1a^f/f^* epithelial endometrial cells. Scale bars: 100 μm. Statistical analysis was performed using one‐way ANOVA analysis followed by the Tukey’s multiple comparison test. (E) Representative images and quantification of BrdU‐positive cells in 3D cultures from *Cre:ER* ^T^ *; Arid1a^+/+^*, *Cre:ER* ^T^ *; Arid1a^f/+^* or *Cre:ER* ^T^ *; Arid1a^f/f^* endometrial epithelial cells. Nuclei were evidenced by Hoechst staining. Scale bars: 25 μm. Statistical analysis was performed using one‐way ANOVA analysis followed by the Tukey’s multiple comparison test. (F) Representative images of western blot analysis of 3D cultures from *Cre:ER* ^T^ *; Arid1a^+/+^*, *Cre:ER* ^T^ *; Arid1a^f/+^* or *Cre:ER* ^T^; *Arid1a^f/f^* epithelial cells showing ARID1A, p16, p21, cyclin D1, cyclin E, cyclin A2, cyclin B, CDK2, CDK4/6 and CDK1 protein expression. GAPDH was used as a loading control. Statistical analysis was performed using one‐way ANOVA analysis followed by the Tukey’s multiple comparison test. (G) Left: representative images showing hematoxylin‐eosin (HE) staining on endometrial sections from *Cre:ER* ^T^ *; Arid1a ^+/+^* (*n* = 10), *Cre:ER* ^T^ *; Arid1a^f/+^* (*n* = 10) or *Cre:ER* ^T^ *Arid1a^f/f^* (*n* = 10) mice. Magnification images of framed regions of the samples are shown. Right: evaluation of endometrial histology in all three different groups after 56–56 weeks post Tamoxifen injection. Scale bars: 100 μm. Statistical analysis was performed using Fisher’s exact test. (H) Representative image (left) and quantification (right) of ki67 immunohistochemistry performed on serial endometrial tissue sections from *Cre:ER* ^T^ *; Arid1a^+/+^*, *Cre:ER* ^T^ *; Arid1a^f/+^* or *Cre:ER^T^; Arid1a^f/f^* mice. Magnification images of framed regions of the samples are shown. Scale bars: 100 μm. Statistical analysis was performed using one‐way ANOVA analysis followed by the Tukey’s multiple comparison test. Graph values are the mean and error bars represented as mean ± SEM; n.s. (not significant *P* ≥ 0.05). Results shown are representative of three independent experiments with three technical replicates per experiment. Graphs showing quantification of immunoblotting plots are shown in Fig. S7; E.V., empty vector.

Because some authors have postulated that ARID1A has a tumour suppressor role, first we reasoned that altered expression of ARID1A would be associated to endometrial tumour initiation. To explore this, we established three‐dimensional (3D) cultures of wild‐type mouse endometrial epithelial cells infected with lentiviruses carrying sgRNA against *Arid1a* (lentiCRISPRv2‐ARID1A.2 and lentiCRISPRv2‐ARID1A.3). Eight to ten days after infection, proliferation rates were analysed by glandular perimeter and BrdU incorporation assays. The results obtained (Fig. 1A,B) did not show any difference between conditions. Next, we examined key regulatory proteins of cell cycle progression (Cyclin D1, Cyclin E, Cyclin A2, Cyclin B, CDK2, CDK4 and CDK1), as well as cell cycle inhibitors (p16 and p21). No differences in the expression levels of cyclins, cyclin‐dependent kinases (CDKs) or cell cycle inhibitors were observed (Fig. 1C). Finally, to strengthen these results, we examined by FACS, the cell cycle profile of endometrial epithelial cells infected with lentiviruses carrying sgRNA against *Arid1a*. As shown in Fig. S1A, we did not observe any change in cell cycle phases in ARID1A downregulated expression cells compared to wild‐type ARID1A parental cells. Furthermore, we evaluated the effect of ARID1A down‐regulation by sgRNA‐ARID1A on HES cells (an extensively used cell line derived spontaneously from primary human endometrial epithelial cells) [22]. As shown in Fig. S1B–E, the downregulation of ARID1A expression did not modify the proliferation rates (cumulative population doublings, analysis of glandular perimeters and BrdU Incorporation assays) or the protein expression levels of cyclins, CDKs or cell cycle inhibitors.

To confirm these results, we established 3D endometrial cultures from mice carrying the tamoxifen‐inducible Cre estrogen receptor recombinase (*Cre:ER* ^T^) [34], and ARID1A‐floxed alleles (*Arid1a^f/+^ or Arid1a^f/f)^)*. As expected, the analysis of glandular perimeters (Fig. 1D), and BrdU incorporation (Fig. 1E) did not reveal substantial differences between *Arid1a^f/+^* and *Arid1a^f/f^* glands. Next, as shown in Fig. 1F, no differences were observed upon evaluating the cell cycle major regulatory protein levels, even when we analysed, by FACS, their cell cycle phase profile (Fig. S1F).

Afterwards, we explored the relevance of ARID1A in tumour initiation *in vivo*. For this purpose, *Cre:ER* ^T^ *; Arid1a^f/f^* or *Cre:ER* ^T^ *; Arid1a^f/+^* mice were intraperitoneally injected with one single dose of tamoxifen, 5 weeks from birth. After 52–56 weeks, mice were sacrificed and ARID1A expression in the uterus was assessed by immunohistochemistry (IHC) (Fig. S1G). Histopathological analysis did not show any gross pathological phenotype in *Cre:ER* ^T^ *; Arid1a^f/f^* or *Cre:ER* ^T^ *; Arid1a^f/+^* mice, as shown in Fig. 1G. In addition, the analysis of the epithelial proliferation marker Ki67 did not reveal relevant changes in proliferation rates in any condition (Fig. 1H).

### 3.2 Loss of ARID1A enhances tumour growth and progression of endometrial cancer cells by impacting the G2/M DNA damage checkpoint

Since a growing body of evidence [17, 18] indicates that downregulation of ARID1A expression could confer adaptive advantages on an established oncological cellular context, we decided to downregulate endogenous ARID1A protein levels in human EEC cell lines.

**Fig. 2:**
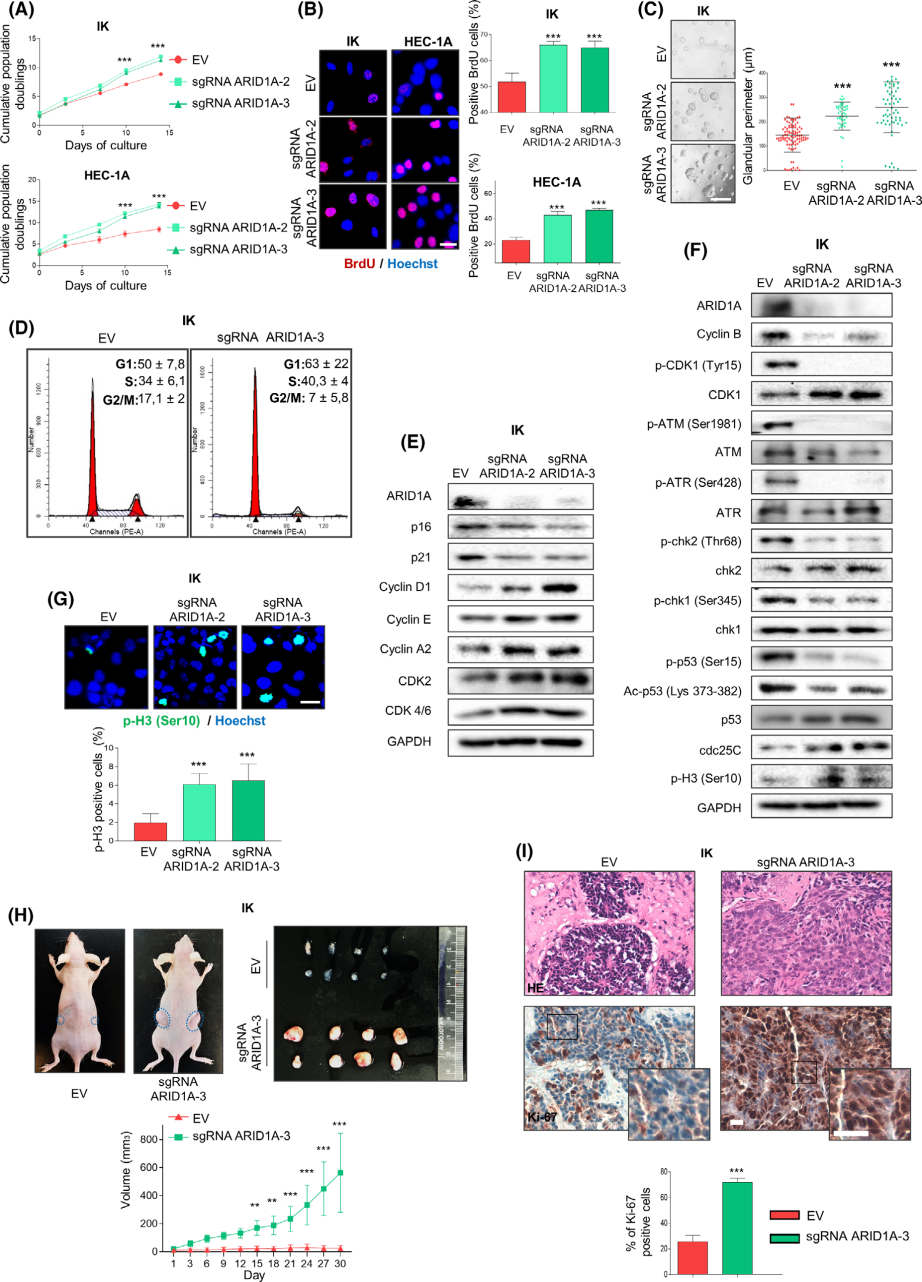
Loss of ARID1A expression in endometrial cancer cell lines enhances tumour growth and progression by a failure in G2/M DNA damage checkpoint. (A) Cumulative population doubling levels of IK and HEC‐1A cells infected with lentiviruses carrying sgRNA against *ARID1A* (lentiCRISPRv2‐ARID1A.2 and lentiCRISPRv2‐ARID1A.3) after 15 days of culture. Data are shown for three independent experiments. Statistical analysis was performed using one‐way ANOVA analysis followed by the Tukey’s multiple comparison test. (B) Representative images and quantification of BrdU‐positive IK and HEC‐1A cells infected with lentiviruses carrying sgRNA against *ARID1A* (lentiCRISPRv2‐ARID1A.2 and lentiCRISPRv2‐ARID1A.3). Nuclei were evidenced by Hoechst staining. Scale bars: 25 μm. Statistical analysis was performed using one‐way ANOVA analysis followed by the Tukey’s multiple comparison test. (C) Representative phase contrast images and measurement of gland perimeter corresponding to 3D cultures of IK cells infected with lentiviruses carrying sgRNA against *ARID1A* (lentiCRISPRv2‐ARID1A.2 and lentiCRISPRv2‐ARID1A.3). Scale bars: 100 μm. Statistical analysis was performed using one‐way ANOVA analysis followed by the Tukey’s multiple comparison test. (D) Representative histograms of cell cycle distribution of IK cells infected or not with lentiviruses carrying sgRNA against *ARID1A* (lentiCRISPRv2‐ARID1A.3). Statistical analysis was performed using paired two‐tailed Student *t*‐test analysis. (E) Representative images of western blot analysis of IK cells infected with lentiviruses carrying sgRNA against *ARID1A* (lentiCRISPRv2‐ARID1A.2 and lentiCRISPRv2‐ARID1A.3) showing ARID1A, p16, p21, Cyclin D1, Cyclin E, Cyclin A2, CDK2 and CDK4/6 protein expression. GAPDH was used as a loading control. (F) Representative images of western blot analysis of IK cells infected with lentiviruses carrying sgRNA against *ARID1A* (lentiCRISPRv2‐ARID1A.2 and lentiCRISPRv2‐ARID1A.3) showing ARID1A, Cyclin B, p‐CDK1 (Tyr15), total CDK1, p‐ATM (Ser1981), p‐ATR (Ser428), p‐chk2 (Thr68), p‐chk1 (Ser345), p‐p53 (Ser15), Ac‐p53 (Lys 373‐382), their total proteins, cdc25C and p‐H3 (Ser10). GAPDH was used as a loading control. (G) Representative images and quantification of p‐histone H3 immunofluorescence stains of IK cells infected with lentiviruses carrying sgRNA against *ARID1A* (lentiCRISPRv2‐ARID1A.2 and lentiCRISPRv2‐ARID1A.3). Scale bars: 25 μm. Nuclei were evidenced by Hoechst staining. Statistical analysis was performed using one‐way ANOVA analysis followed by the Tukey’s multiple comparison test. (H) Subcutaneous xenografts from IK cells infected with lentiviruses carrying sgRNA against *ARID1A* (lentiCRISPRv2‐ARID1A.3) (*n* = 8) and IK control cells (*n* = 8). After engraftment, tumour growth was tracked daily for 30 days. Upper panel, representative images of subcutaneous xenografts. Bottom, plot from tumour growth kinetics showing by tumour volume (mm^3^) Statistical analysis was performed using paired two‐tailed Student *t*‐test analysis. (I) Upper panel, representative images showing hematoxylin‐eosin (HE) and Ki67 immunostaining on xenograft tumours from IK cells infected with lentiviruses carrying sgRNA against *ARID1A* (lentiCRISPRv2‐ARID1A.3) or control (empty vector) IK cells. Bottom, quantification of ki67 positive cells. Scale bars: 100 μm. Statistical analysis was performed using paired two‐tailed Student *t*‐test analysis. Graph values are the mean and error bars represented as mean ± SEM. **P < 0.01; ***P < 0.001. Results shown are representative of three independent experiments with three technical replicates per experiment. Graphs showing quantification of immunoblotting plots are shown in Fig. S7; E.V., empty vector.

A panel of human EEC cell lines was evaluated to analyse the endogenous levels of ARID1A (Fig. S2A). Next, Ishikawa (IK), MFE‐296 and HEC‐1‐A EEC cell lines, expressing wild‐type levels of ARID1A, were infected with lentiviruses carrying sgRNAs against ARID1A. ARID1A downregulation increased proliferation rates of EEC cells, as revealed by the cumulative population doubling levels, BrdU‐incorporation and 3D glandular assays (Fig. 2A–C and Fig. S2B–D). Moreover, we analysed, by FACS, the cell cycle profile of those cells, to reject the possibility that ARID1A downregulated cells were in G2/M checkpoint arrest (Fig 2D). Consistent with these findings, we detected decreased protein levels of cell cycle inhibitors p16 and p21 and increased levels of cyclins D1, E and A2, and CDK2 and CDK4/6 in all ARID1A knockdown cells compared to wild‐type ARID1A parental cells (Fig. 2E and Fig. S2E).

Cyclin B accumulation occurs after G2‐to‐M transition when there is high CDK1‐CyclinB kinase activity. Once mitosis is initiated, cyclin B degradation occurs to promote mitotic exit [35]. When we analysed cyclin B levels in ARID1A knockdown cells, we observed a decreased level expression (Fig. 2F). However, when we analysed the activation of CDK1‐CyclinB complex, we observed an increased expression of CDK1 total protein levels and a concurrent decreased rank expression of inactivating CDK1‐Tyrosine 15 phosphorylation (Fig. 2F and Fig. S2F). Considering that the CDK1‐cyclin B complex has an important role in G2‐to‐M cell cycle transition [35], we decided to analyse the status of G2/M cell cycle checkpoint regulators in control and ARID1A knockdown cells by western blot. Our results showed that ARID1A downregulation is associated with decreased activation of G2/M cell cycle checkpoint promoters such as Chk2, Chk1 and p‐p53. Furthermore, a concomitant increase in the protein levels of Cdc25C, a G2/M negative regulator checkpoint, was seen (Fig. 2F). Consistently, increased phosphorylation of histone H3 at Serine 10 suggested that decreased levels of ARID1A promote G2‐to‐M progression in EEC cells (Fig. 2F,G). Altogether, these results showed that ARID1A loss of expression can promote omission of G2/M cell cycle arrest imposed by oncogene‐induced replicative stress, leading to genomic instability.

Finally, in order to determine the effects of ARID1A downregulation on tumour growth *in vivo*, we subcutaneously engrafted ARID1A knockdown and their parental IK cells in immunodeficient mice (NSG), to generate subcutaneous tumours. As shown in Fig. 2H ARID1A, downregulated tumours were larger compared to parental cell xenograft tumours. Finally, at the molecular level, ARID1A knockdown expression xenografts showed a substantial increase in the expression of the Ki67 proliferation marker compared with ARID1A wild‐type expression xenografts (Fig. 2I).

### 3.3 ARID1A down expression promotes EMT in endometrial cancer context

**Fig. 3:**
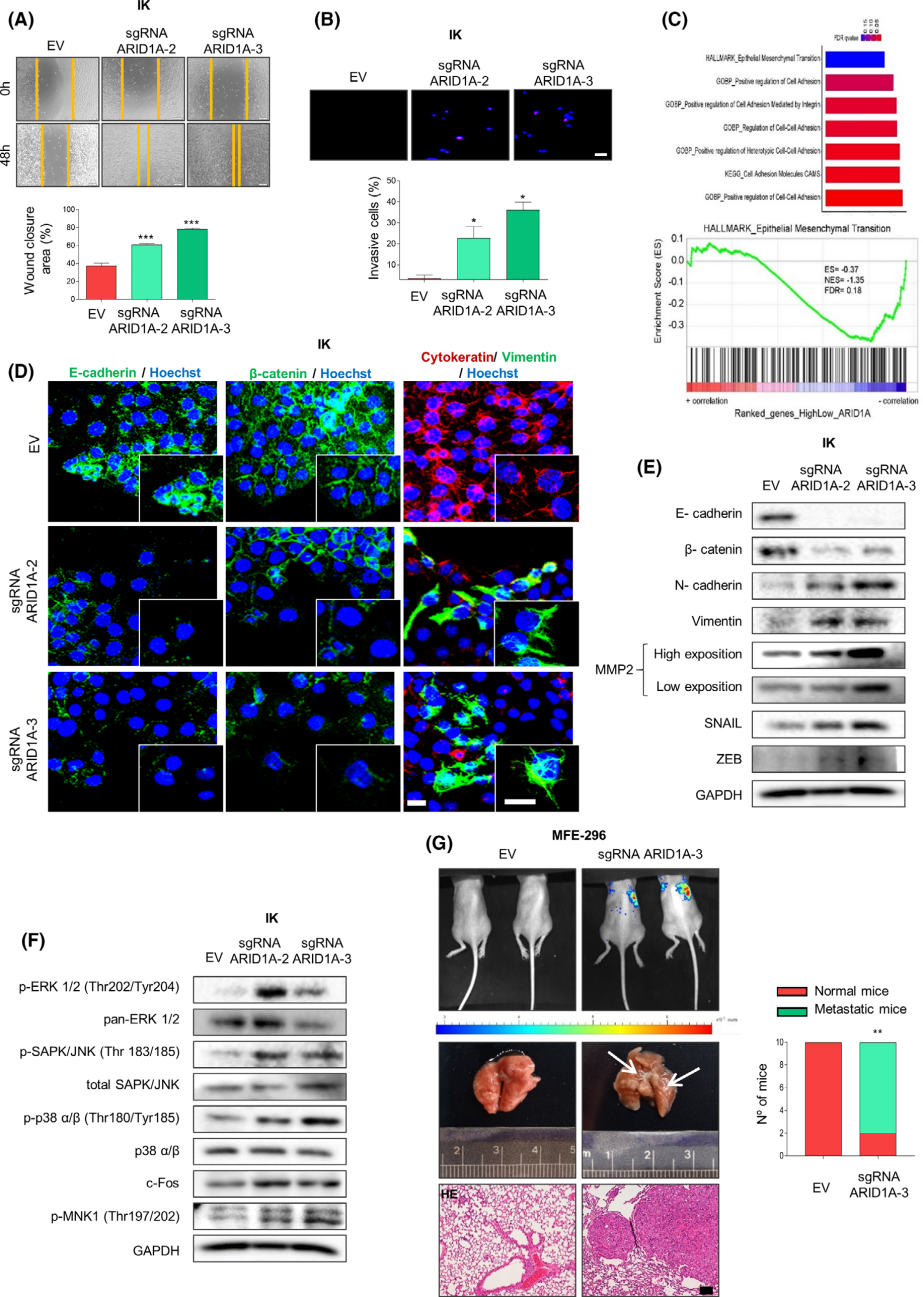
ARID1A down‐expression promotes EMT process in endometrial cancer context. (A) Representative images at time 0 and 48 h after scratch, of wound‐healing assay performed in IK cells infected with lentiviruses carrying sgRNA against *ARID1A* (lentiCRISPRv2‐ARID1A.2 and lentiCRISPRv2‐ARID1A.3) (top panel) and quantification of wound closure area between the indicated time (bottom blot). Statistical analysis was performed using one‐way ANOVA analysis followed by the Tukey’s multiple comparison. (B) Representative images of nuclear Hoechst staining of transwell invasion assay after the cotton swab in IK cells infected with lentiviruses carrying sgRNA against *ARID1A* (lentiCRISPRv2‐ARID1A.2 and lentiCRISPRv2‐ARID1A.3) (upper panel) and quantification of Matrigel^®^ invasive cells (bottom plot). Scale bars: 50 μm. Statistical analysis was performed using one‐way ANOVA analysis followed by the Tukey’s multiple comparison. (C) Gene set enrichment analysis (GSEA) was used to uncover signatures associated to cellular adhesion and EMT (Upper) and enriched gene (bottom) from the molecular signatures database TCGA_UCEC. (D) Representative images of immunofluorescence against E‐cadherin, β‐catenin, Cytokeratin, Vimentin and Hoechst in IK cells infected with lentiviruses carrying sgRNA against *ARID1A* (lentiCRISPRv2‐ARID1A.2 and lentiCRISPRv2‐ARID1A.3). Magnification images of framed regions of the samples are shown. Scale bars: 25 μm. (E) Representative immunoblots showing E‐cadherin, β‐catenin, N‐cadherin, Vimentin, MMP2, SNAIL and ZEB protein expression in IK cells infected with lentiviruses carrying sgRNA against *ARID1A* (lentiCRISPRv2‐ARID1A.2 and lentiCRISPRv2‐ARID1A.3). GAPDH was used as a loading control. (F) Western blot analysis of phosphorylated and total ERK 1/2, SAPK/JNK and p38 α/β, c‐fos and phospho‐MNK1 in IK cells infected with lentiviruses carrying sgRNA against *ARID1A* (lentiCRISPRv2‐ARID1A.2 and lentiCRISPRv2‐ARID1A.3). GAPDH was used as a loading control. (G) Metastatic growth of stably infected with EGFP‐luciferase MFE‐296 cells infected with lentiviruses carrying sgRNA against *ARID1A* (lentiCRISPRv2‐ and lentiCRISPRv2‐ARID1A.3). After selection, 50 × 10^4^ MFE‐296 cells were retro‐orbitally injected and let grow for 10 days (see the Materials and methods section for further details). Left‐up: representative bioluminescence imaging comparing metastasis in lungs 10 days after injection of MFE‐296 ARID1A deficient cells and the parental MFE‐296 cell line. Left‐down: representative lung and lung morphology analysis by hematoxylin and eosin staining (HE). Right, representative graph evaluating lung histology lesions present in indicated group animals. Scale bars: 100 μm. Statistical analysis was performed using fisher’s exact test. Graph values are the mean and error bars represented as mean ± SEM. *P < 0.05; **P < 0.01; ***P < 0.001. Results shown are representative of three independent experiments with three technical replicates per experiment. Graphs showing quantification of immunoblotting plots are shown in Fig. S7; E.V., empty vector.

Recent studies have evidenced that *ARID1A* mutations are predominantly subclonal, heterogeneous and may alter the epigenetic landscape to foster cancer cell dissemination of already established tumours. Interestingly, we noticed that altered expression of ARID1A was associated with an altered morphological cell appearance. Specifically, we found that the parental EC cell lines grew in clustered colonies with high cell‐cell adhesion, while cells with down‐expressed ARID1A protein levels grew spreading with elongated, spindle‐shaped morphologies and frequent filopodia (Fig. S3A). On the basis of these lines of evidence, we next assessed the effect of ARID1A down‐expression in cell migration and invasion. As predicted, downregulation of ARID1A increased migration and invasiveness capabilities as shown by wound healing migration and transwell invasion assays (Fig. 3A,B and Fig. S3B,C).

In order to identify a potential mechanistic basis to explain the effects of ARID1A knockdown, we decided to explore the genetic program associated with decreased ARID1A expression. To this end, we performed GSEA on RNA sequencing data obtained from the TCGA_UCEC database [32]. Specifically, and in line with the observation that ARID1A mutations are found enriched in type‐I endometrial tumors, we focused our analysis on TCGA_UCEC tumors of endometrioid histology. We compared the 20% of samples presenting the highest ARID1A expression with the lowest 20%. Remarkably, we found that ARID1A expression is inversely correlated with signatures associated to cellular adhesion and epithelial to mesenchymal transition (Fig. 3C). It is well known that EMT changes are central processes in migration and invasion phenomena of endometrial neoplastic cells [36]. Thus, to further investigate the role of ARID1A in the regulation of EMT phenotype, we performed immunofluorescence assays to study the expression and localization of several epithelial and mesenchymal markers. The results obtained showed that ARID1A knockdown cells present a down‐expression of epithelial protein markers such as E‐cadherin, β‐catenin and cytokeratin and an upregulation of the mesenchymal protein marker vimentin (Fig. 3D and Fig. S3E). Similar results were obtained by immunoblotting assay, when we evaluated several epithelial (E‐cadherin and β‐catenin) and mesenchymal (Vimentin, N‐cadherin and MMP2) markers. Furthermore, upregulation of SNAIL1 and ZEB (transcriptional factors that regulates EMT process [37]) were detected upon ARID1A‐knockdown expression (Fig. 3E and Fig. S3F).

It is widely accepted that the MAPK/ERK signalling pathway is present in the regulation of migratory and invasiveness capabilities of endometrial cancer cells [38, 39]. Next, we asked whether this molecular pathway could be over‐activated in ARID1A‐deficient cells. For this purpose, we evaluated the phosphorylation and total protein levels of MAPK/ERK pathway proteins effectors (ERK1/2, SAPK/JNK, p38 α/β, c‐fos and MNK1) by immunoblotting assay. As displayed in Fig. 3F and Fig. S3G, we observed that the MAPK/ERK pathway was substantially activated by the ARID1A downregulation.

Next, to explore the role of ARID1A in cell migration, invasion and dissemination *in vivo*, we investigated the ability of ARID1A defective MFE‐296 cells to promote metastatic lung colonization using a retro‐orbital injection assay previously reported [25]. Remarkably, as shown in Fig. 3G, MFE‐296 ARID1A defective expression fostered a dramatic lung metastatic spread when compared to MFE‐296 control cells, which were unable to metastasise or grow 10 days after the injection.

### 3.4 ARID1A deficiency omits DSB DNA damage apoptotic response induced by etoposide treatment

**Fig. 4:**
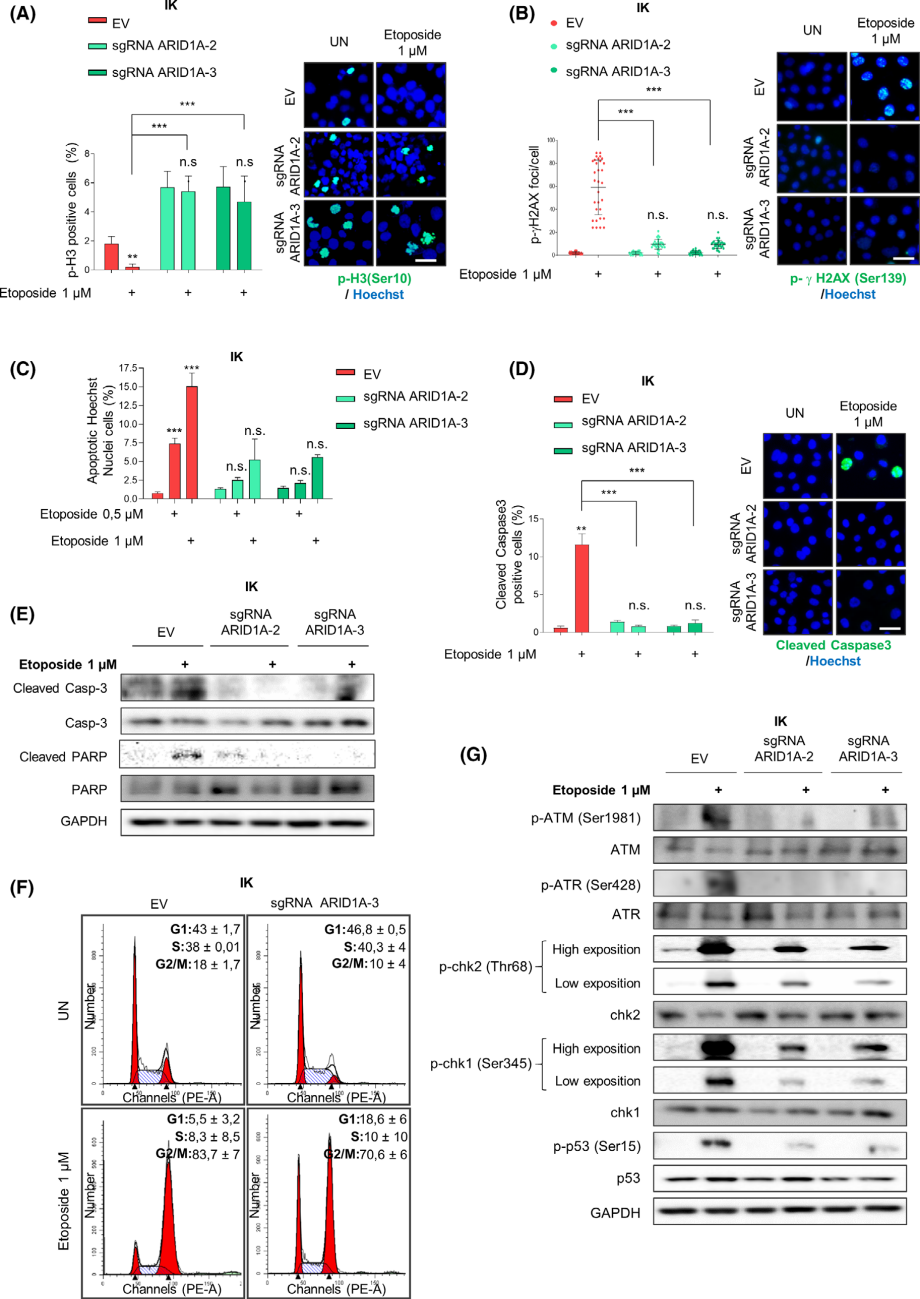
ARID1A deficiency omits DSB DNA damage apoptotic response induced by etoposide treatment. IK cells infected with lentiviruses carrying sgRNA against *ARID1A* (lentiCRISPRv2‐ARID1A.2 and lentiCRISPRv2‐ARID1A.3) treated or not with etoposide 1 µm during 36h. (A) (Left) Quantification and (right) representative images of cells stained with p‐Histone H3 and Hoechst to show mitotic cells under the indicated conditions. Scale bars: 25 μm. Statistical analysis was performed using two‐way ANOVA analysis followed Bonferroni *post hoc* analysis. (B) Right panel shows representative images of cell cultures immunofluorescence against p‐histone γH2AX and Hoechst to show DNA damage in cells under the indicated conditions. Left plot indicates quantification of positive staining for p‐γH2AX foci/cell in cells exposed to the described treatments. Scale bars: 25 μm. Statistical analysis was performed using two‐way ANOVA analysis followed Bonferroni *post hoc* analysis. (C) Quantification of cells displaying apoptotic nuclei morphologies exposed by Hoechst staining under conditions indicated. Statistical analysis was performed using two‐way ANOVA analysis followed Bonferroni *post hoc* analysis. (D) Representative images and quantification of immunostaining for Cleaved Caspase‐3. Nuclei were evidenced by Hoechst staining. Scale bars: 25 μm. Statistical analysis was performed using two‐way ANOVA analysis followed Bonferroni *post hoc* analysis. (E) Representative immunoblots showing cleaved and total Caspase‐3 and PARP protein expression. GAPDH was used as a loading control. (F) Representative histograms of cell cycle distribution. (G) Representative immunoblots showing phosphorylated ATM (Ser1981), ATR (Ser428), chk2 (Thr68), chk1 (Ser345) and p‐p53 (Ser15), and total protein expressions. GAPDH was used as a loading control. Graph values are the mean and error bars represented as mean ± SEM. **P < 0.01; ***P < 0.001, n.s. (not significant *P* ≥ 0.05). Results shown are representative of three independent experiments with three technical replicates per experiment. Graphs showing quantification of immunoblotting plots are shown in Fig. S7; E.V., empty vector.

In cells, the Ataxia‐telangiectasia mutated/Ataxia telangiectasia and Rad3‐related protein (ATM/ATR‐regulated) DNA damage response pathway activate cell cycle checkpoints or apoptosis upon various endogenous or exogenous DNA damage stimuli [40]. Given the observed defective G2/M cell cycle checkpoint activation and taking into account its central role in DNA double strand breaks (DSBs) repair [41], we decided to evaluate if ARID1A loss of expression could omit DSB apoptotic response in EC cells. For this purpose, we treated our ARID1A‐deficient cells with etoposide, an inducer of DSBs that triggers cell cycle arrest and apoptosis through p53 activation [42]. Afterwards, we analysed G2‐to‐M transition using phospho‐Histone H3 immunofluorescence. After exposure to etoposide, ARID1A‐wildtype cells showed a significant reduction in the percentage of mitotic cells (p‐H3 positive cells). Conversely, ARID1A‐altered cells exhibited no differences in mitotic percentages after etoposide treatment (Fig. 4A), suggesting a defective G2/M checkpoint activation. Next, to further characterise this cellular response, we analysed the levels of үH2AX phosphorylation, a canonical marker of DNA damage response, and the apoptotic commitment of these cells after etoposide treatment. Surprisingly, while the number of p‐үH2AX foci per cell increased significantly in etoposide‐treated control cells, ARID1A‐deficient cells presented no significant differences after treatment (Fig. 4B and Fig. S4A). Moreover, as shown in Fig. 4C,D and Fig. S4B,C, etoposide treatment induced a consistent apoptotic response in parental cell lines but not in ARID1A defective cells. Accordingly, analysis of protein levels of cleaved caspase‐3 and PARP evidenced that etoposide was not able to activate an apoptotic effector program in cells with altered ARID1A expression (Fig. 4E and Fig. S4D).

In order to analyse, the effects of etoposide treatment in cell cycle profile, we performed a FACS analysis of ARID1A‐deficient and ARID1A‐wild‐type cells. As evidenced in Fig. 4F, G2‐M checkpoint arrest induced by etoposide treatment was enhanced in ARID1A wild‐type cells compared to sgRNA ARID1A cells. This result reinforces the hypothesis that decrease in ARID1A expression decreases DSB apoptotic response to etoposide treatment in EC cells.

Since one of major DSB cellular responses is regulated by ATM/ATR‐cascade promoting homologous recombination (HR) DNA repair or apoptosis induction [43], we examined, using immunoblotting assays, the protein levels of the downstream effectors of this pathway. In agreement with our previous results, we did not observe a substantial increase in total levels of phosphorylated ATM, ATR, Chk1, Chk2 and p53 in ARID1A‐defective cells after DSB induction, as opposed to control cells (Fig. 4G and Fig. S4E).

### 3.5 Loss of ARID1A expression promotes HDAC6 upregulation in EC

**Fig. 5:**
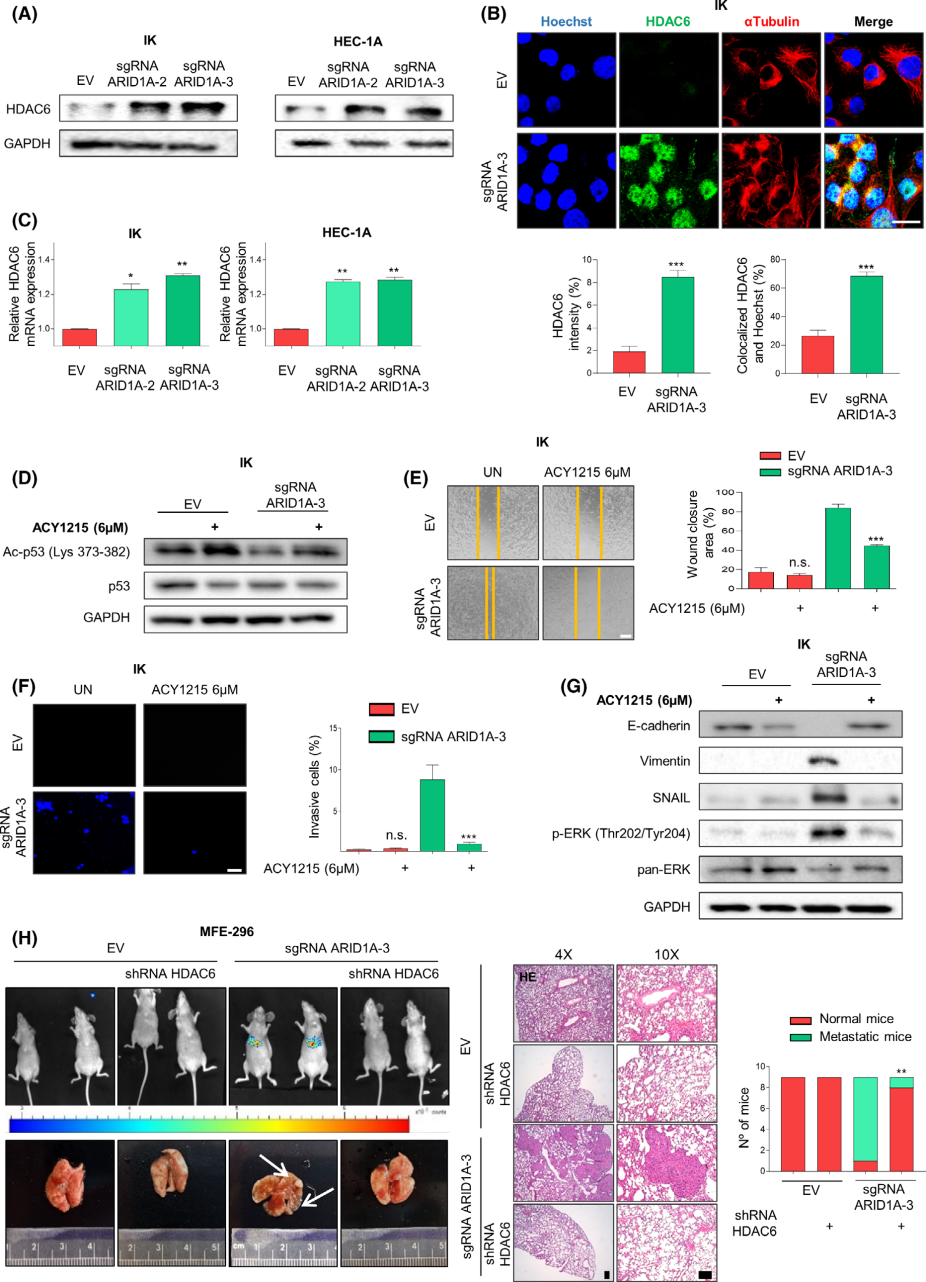
Loss of ARID1A expression promotes HDAC6 upregulation in EC. (A) Western blot analysis of HDAC6 expression in IK and HEC‐1A cells infected with lentiviruses carrying sgRNA against *ARID1A* (lentiCRISPRv2‐ARID1A.2 and lentiCRISPRv2‐ARID1A.3). GAPDH was used as a loading control. (B) Upper panel shows representative images of HDAC6 immunofluorescence performed in IK cells infected with lentiviruses carrying sgRNA against *ARID1A* (lentiCRISPRv2‐ARID1A.3). Tubulin antibodies and Hoechst were used to show cell nuclei and cytoskeleton. Bottom blots show quantification of HDAC6 immunofluorescence intensity (left blot) and HDAC6 colocalization with the nuclear marker Hoechst (right blot). Scale bars: 25 μm. Statistical analysis was performed using Student *t*‐test analysis. (C) RT‐qPCR of HDAC6 mRNA expression in IK and HEC‐1A cells infected with lentiviruses carrying sgRNA against *ARID1A* (lentiCRISPRv2‐ARID1A.2 and lentiCRISPRv2‐ARID1A.3). Statistical analysis was performed one‐way ANOVA analysis followed by the Tukey’s multiple comparison test. (D) Representative immunoblotting images of Ac‐p53 (Lys 373‐382) and total p53 protein expression in IK cells infected with lentiviruses carrying sgRNA against *ARID1A* (lentiCRISPRv2‐ARID1A.3) and control cells, untreated or treated with ACY‐1215 6 µm for 48 h. GAPDH was used as a loading control. (E) Representative images 48 h after scratch (left), of wound‐healing assay performed in IK cells infected with lentiviruses carrying sgRNA against *ARID1A* (lentiCRISPRv2‐ARID1A.2 and lentiCRISPRv2‐ARID1A.3) and control cells, untreated or treated with ACY‐1215 6 µm for 48 h. Right blot shows quantification of wound closure area between the indicated time. Scale bars: 200 μm. Statistical analysis was performed using two‐way ANOVA analysis followed Bonferroni *post hoc* analysis. (F) Representative images of nuclear Hoechst staining of transwell invasion assay after the cotton swab in IK cells infected with lentiviruses carrying sgRNA against *ARID1A* (lentiCRISPRv2‐ARID1A.2 and lentiCRISPRv2‐ARID1A.3) and control cells untreated or treated with ACY‐1215 6 µm for 48 h (left panel), and quantification of Matrigel^®^ invasive cells (right plot). Scale bars: 50 μm. Statistical analysis was performed using two‐way ANOVA analysis followed Bonferroni *post hoc* analysis. (G) Western blot analysis of E‐cadherin, Vimentin, SNAIL, phosphor‐ERK (Thr202/Tyr204) and total pan‐ERK protein expression in IK cells infected with lentiviruses carrying sgRNA against *ARID1A* (lentiCRISPRv2‐ARID1A.2 and lentiCRISPRv2‐ARID1A.3) and control cells untreated or treated with ACY‐1215 6 µm for 48 h. (H) Metastatic growth of stably infected with EGFP‐luciferase MFE‐296 ARID1A deficient (or not) cells transduced with lentiviral particles containing shRNA‐scrambled or shRNA‐HDAC6. After selection, 50 × 10^4^ MFE‐296 cells were retro‐orbitally injected and let grow for 10 days (see the Materials and methods section for further details). Left, representative bioluminescence imaging comparing metastasis in lungs 10 days after the injection of MFE‐296 ARID1A deficient cells and the parental MFE‐296 cell line. Representative macroscopic lung images. Middle Representative lung morphology analysis by haematoxylin and eosin staining (HE). Samples were harvested at day 10 based on bioluminescent analysis. Right: quantification of lung histology lesions present in indicated group animals. Scale bars: 100 μm. Statistical analysis was performed using Fisher’s exact test. Graph values are the mean and error bars represented as mean ± SEM. *P < 0.05, **P < 0.01; ***P < 0.001, n.s. (not significant *P* ≥ 0.05). Results shown are representative of three independent experiments with three technical replicates per experiment. Graphs showing quantification of immunoblotting plots are shown in Fig. S7; E.V., empty vector.

Recently, it has been described that ARID1A is able to transcriptionally repress histone deacetylase 6 (HDAC6) in ovarian clear cell carcinoma (OCCC); and when loss of ARID1A expression occurs, suppression of HDAC6 is relieved [44]. Moreover, HDAC6 expression is increased in several cancer types, and it has been associated with poor prognosis EC [45]. Taking these studies into account, we decided to examine the association between ARID1A and HDAC6 levels in EC. First, we assessed HDAC6 expression levels in ARID1A‐deficient cells compared with parental ARID1A‐wild‐type cells. As shown in Fig. 5A,B, HDAC6 was significantly overexpressed in sgRNA‐ARID1A cells compared with control cells. Moreover, in the immunofluorescence assay (Fig. 5B), we observed that HDAC6 localization was predominantly nuclear, which has been previously suggested to trigger an EMT process [46].

Further, we evaluated HDAC6 mRNA expression in our paired cell lines. qRT‐PCR analysis revealed that ARID1A‐knockdown cell lines present significantly increased mRNA levels of HDAC6 compared with ARID1A‐wild type (Fig. 5C), suggesting that in EC cell lines, ARID1A may directly repress transcription of HDAC6.

To examine the role of HDAC6 in the context of ARID1A‐perturbed expression cells, we tested the selective and specific HDAC6 inhibitor ACY1215 (Ricolinostat) in our cells. First, to confirm that HDAC6 function was inhibited by ACY1215, we evaluated, using an immunoblotting assay, the levels of p53 acetylation in lysine 373/382, a direct substrate of HDAC6 [47]. As expected, ACY1215 treatment restored acetylation levels of p53 in ARID1A altered cells (Fig. 5D and Fig. S5A).

As explained before, a novel nuclear epigenetic role has been reported for HDAC6 in EMT process, inducing cell dedifferentiation [46]. Our previous data (Fig. 5B) encouraged us to investigate whether inhibition of HDAC6 expression could revert the migration and invasive phenotype of ARID1A knockdown cells. By performing wound healing and transwell invasion assays in IK and HEC‐1A ARID1A‐paired cell lines, we observed that pharmacological inhibition of HDAC6 activity suppressed migratory and invasive capacities promoted by loss of ARID1A expression (Fig. 5E,F and Fig. S5B,C). Furthermore, as shown in Fig. 5G and Fig. S5D, exposure of ARID1A‐knockdown cells to ACY1215 restored E‐cadherin protein levels while decreasing vimentin and SNAIL EMT markers. Interestingly, this effect was accompanied by a restoration of basal ERK1/2 phosphorylation levels.

Next, we aimed to replicate these results *in vivo*. To this end, we first transduced control and sgRNA‐ARID1A MFE‐296 cells with lentiviral particles containing a control shRNA or shRNA against HDAC6 (Fig. S5E); afterwards, we evaluated the migratory and invasive capability of those cells in order to confirm that shRNA‐HDAC6 transduction recapitulated the phenotype seen after ACY1215 treatment. As seen in Fig. S5F,G, downregulation of HDAC6 expression using shRNA also reverted the migratory and invasive abilities promoted by the decrease of ARID1A expression. Subsequently, we retro‐orbitally injected EGFP/luciferase‐MFE‐296 ARID1A‐deficient expression and their parental cell line transduced with shRNA‐scrambled or shRNA‐HDAC6 lentivirus into NSG mice. After 10 days, we examined whether HDAC6 downregulation could affect the lung EC metastatic growth. Strikingly, while metastases of ARID1A‐deficient cells grew unopposed, ARID1A‐deficient cells transduced with shRNA‐HDAC6 did not present any metastatic foci (Fig. 5H).

### 3.6 Resistance to DSB‐induced apoptosis upon etoposide treatment in cells with ARID1A loss expression is reversed by ACY1215 treatment

In mammalian cells, DNA DSBs are predominantly repaired by HR and non‐homologous end‐joining (NHEJ) pathways. The balance between both pathways is essential for genome stability, and disturbances on one of those pathways often leads to oncologic transformation or to the acquisition of the malignant phenotype [48]. As shown previously in Fig. 4, ARID1A inactivation induced a negative regulation of HR repair pathway. However, it remains unclear which DNA repair or molecular pathway is employed by ARID1A‐deficient cells for survival and maintenance of DNA integrity in the face of DSB DNA damage.

Further, some studies have revealed an important role for HDAC6 in the DNA damage repair pathway through deacetylation of KU70, a key player of NHEJ pathway [49, 50, 51]. In light of these observations and taking into account that ARID1A‐deficient cells overexpress HDAC6, we reasoned that HDAC6 inhibition might cause etoposide‐induced apoptosis in ARID1A‐altered cells.

**Fig. 6:**
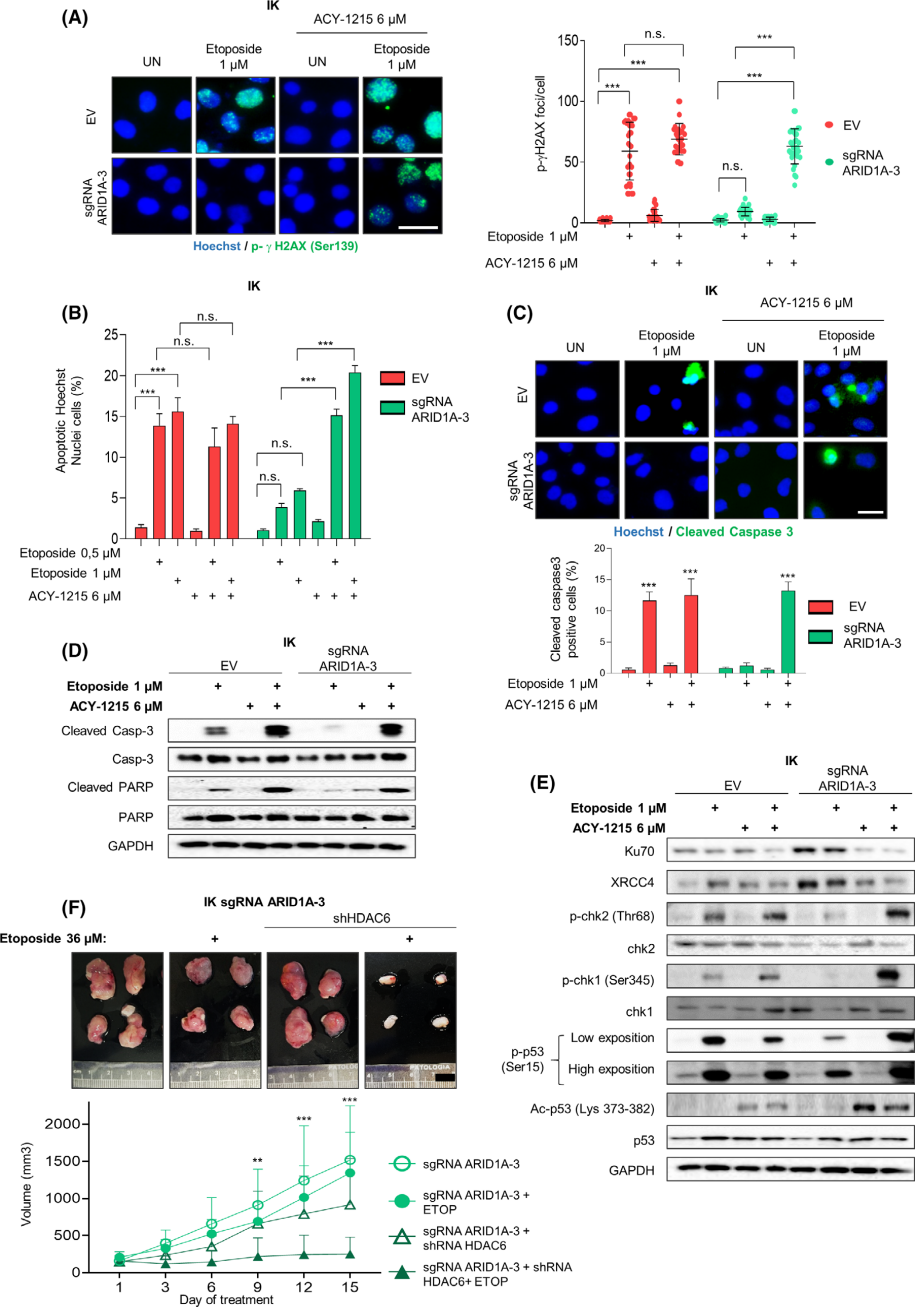
Resistance to DSB‐induced apoptosis promoted by ARID1A loss is reverted with ACY1215. (A) IK cells infected with lentiviruses carrying sgRNA against *ARID1A* (lentiCRISPRv2‐ARID1A.3) treated or not with etoposide 1 µm and/or ACY‐1215 6 µm, for 36 h. Left panel shows representative images of cell cultures immunofluorescence against p‐histone γH2AX and Hoechst to show DNA damage in cells under the indicated conditions. Right plot indicates quantification of positive staining for p‐γH2AX foci/cell in cells exposed to the described treatments. Scale bars: 25 μm. Statistical analysis was performed using two‐way ANOVA analysis followed Bonferroni *post hoc* analysis. (B) Quantification of cells displaying apoptotic nuclei morphologies exposed by Hoechst staining under the same conditions. Statistical analysis was performed using two‐way ANOVA analysis followed Bonferroni *post hoc* analysis. (C) Representative images (upper panel) and quantification (bottom plot) of immunostaining for Cleaved Caspase‐3 in IK cells infected with lentiviruses carrying sgRNA against *ARID1A* (lentiCRISPRv2‐ARID1A.3) and control cells untreated or treated with etoposide 1 µm, ACY‐1215 6 µm or combination of both, for 36 h. Nuclei were evidenced by Hoechst staining. Scale bars: 25 μm. Statistical analysis was performed using two‐way ANOVA analysis followed Bonferroni *post hoc* analysis. (D) Western blot analysis of cleaved and total caspase 3 and PARP protein expression in IK cells infected with lentiviruses carrying sgRNA against *ARID1A* (lentiCRISPRv2‐ARID1A.3) treated or not with etoposide 1 µm and or ACY‐1215 6 µm for 36 h. GAPDH was used as a loading control. (E) Western blot analysis of Ku70, XRCC4, phospho‐chk2 (Thr68), phospho‐chk1 (Ser345), phosphor‐p53 (Ser15), Acetil‐p53 (Lys 373‐382) and total protein expressions in IK cells infected with lentiviruses carrying sgRNA against *ARID1A* (lentiCRISPRv2‐ARID1A.3) treated or not with etoposide 1 µm, ACY‐1215 6 µm or combination of both for 36 h. GAPDH was used as a loading control. (F) Subcutaneous xenografts from IK cells infected with lentiviruses carrying sgRNA against *ARID1A* (lentiCRISPRv2‐ARID1A.3) and transfected with HDAC6 shRNA. After tumour engraftment, etoposide (ETOP) treatments (36 µm) were administrated during 15 days. Tumour growth was tracked daily. Upper panel: representative images of subcutaneous xenografts. Lower panel: plot from tumour growth kinetics showing by tumour volume(mm^3^). Scale bars: 1cm. Statistical analysis was performed using two‐way ANOVA analysis followed Bonferroni *post hoc* analysis. Graph values are the mean and error bars represented as mean ± SEM. **P < 0.01; ***P < 0.001, n.s. (not significant *P* ≥ 0.05). Results shown are representative of three independent experiments with three technical replicates per experiment. Graphs showing quantification of immunoblotting plots are shown in Fig. S7; E.V., empty vector.

To this end, we performed an immunofluorescence assay against phosphorylated үH2AX in ARID1A‐dowregulated expression and parental cells treated with etoposide and ACY1215. The results obtained show that combining etoposide and ACY1215 treatments significantly increases the number of p‐үH2AX foci per cell in ARID1A‐defective cells, in contrast to what we observed in ARID1A deficient cells treated only with etoposide (Fig. 6A and Fig. S6A).

Next, we evaluated the apoptosis status in ARID1A‐perturbed expression cells after the combinatory treatment (etoposide plus ACY1215). As shown in Fig. 6B,C and Fig. S6B,C, ARID1A knockdown‐cells significantly increase the percentage of apoptotic and positive cleaved caspase‐3 cells after etoposide and ACY1215 treatments, compared to ARID1A‐deficient cells treated with etoposide or ACY1215 alone. Furthermore, we analysed, using an immunoblotting assay, the levels of cleaved caspase‐3 and PARP (Fig. 6D and Fig. S6D). Our results showed an increase in the expression of all these pro‐apoptotic proteins in ARID1A‐altered cells treated with etoposide plus ACY1215, thus reverting the etoposide apoptotic resistance of ARID1A deficiency cells.

Next, we sought to identify which specific molecular mechanisms were involved in the acquired vulnerability to DSB damage in ARID1A‐defective cells after etoposide and ACY1215 combinational treatment. We hypothesised that HDAC6 inhibition might disturb the balance between HR/NHEJ DNA repair pathway. To this end, we analysed the main HR pathway effectors: p‐chk1 and p‐p53 and the NHEJ pathway effectors: Ku70 and XRCC4. As shown in Fig. 6E (and Fig. S6E), although ARID1A deficient cells present a steady‐state level of activation of the NHEJ DNA repair pathway (seen by increased levels of Ku70 and XRCC4, even in untreated conditions), they present decreased levels of Ku70 and XRCC4 and increased activating phosphorylation levels of chk1 and p53 after ACY1215 exposure, leading to apoptosis. These results were also confirmed by HDAC6 shRNA transfection (Fig. S6F,G). Finally, in order to determine the effect of etoposide treatment on HDAC6 shRNA‐transfected ARID1A‐defective cells *in vivo*, we subcutaneously engrafted sgRNA ARID1A cells transfected or not with shRNA HDAC6. As evidenced in Fig. 6F, HDAC6 downregulated expression tumours were smaller than HDAC6 wild‐type expression tumours after etoposide treatment. Altogether, these results indicate that inhibition of HDAC6 cause apoptotic cell vulnerability to the DSBs inducer etoposide treatment after ARID1A loss of expression.

## 4 Discussion

In this study, first we demonstrated that ARID1A depletion is not sufficient to initiate endometrial tumorigenesis, although *ARID1A* has been described as a driver gene in colon [52] or ovarian [44] neoplasms. Consequences of loss of ARID1A expression in endometrial epithelium were evaluated by *3D ex‐vivo* endometrial cultures and Cre:ER^T^; Arid1a^f/f^ or Cre:ER^T^; Arid1a^f/+^ mice and no gross differences were observed in terms of cellular proliferation rates, regulatory cell cycle progression protein expression or increased appearance of simple hyperplasia. These results corroborated the recent findings described by Wilson et al. [53] and also reported in an *Arid1a* specific knockout hepatocellular carcinoma mice model [54] and in a colon cancer model [52], where all mice models show that the capacity of ARID1A to develop tumorigenesis is dependent on the presence or absence of co‐occurring mutations. Thus, in certain contexts, ARID1A might function in an oncogenic capacity to support tumour growth.

*ARID1A* has been described as a ‘gatekeeper’ gene, controlling cellular proliferation, usually by regulating cell cycle or promoting apoptosis [55]. Progression through the cell cycle is promoted by CDKs, which are regulated positively by cyclins and negatively by cell cycle inhibitor proteins, and monitored by checkpoints such as Chk2, Chk1 and p53 [56]. Our functional studies clearly showed that depletion of ARID1A increased cellular proliferation in *in vitro* and *in vivo* assays. Furthermore, our results suggested that the observed increased proliferation rates are caused by a major defect in the early G2/M checkpoint activation in the context of cancer, leading in ARID1A‐deficient EC cells to impaired cell cycle arrest, which, under non‐pathological conditions, should delay cell cycle progression to allow the resolution of mutations or DNA damage. This observation suggests that *ARID1A* may also function as a ‘caretaker’, playing an important role in the ATM/ATR‐mediated DNA damage checkpoint, which determines cell fate after DNA damage.

**Fig. 7:**
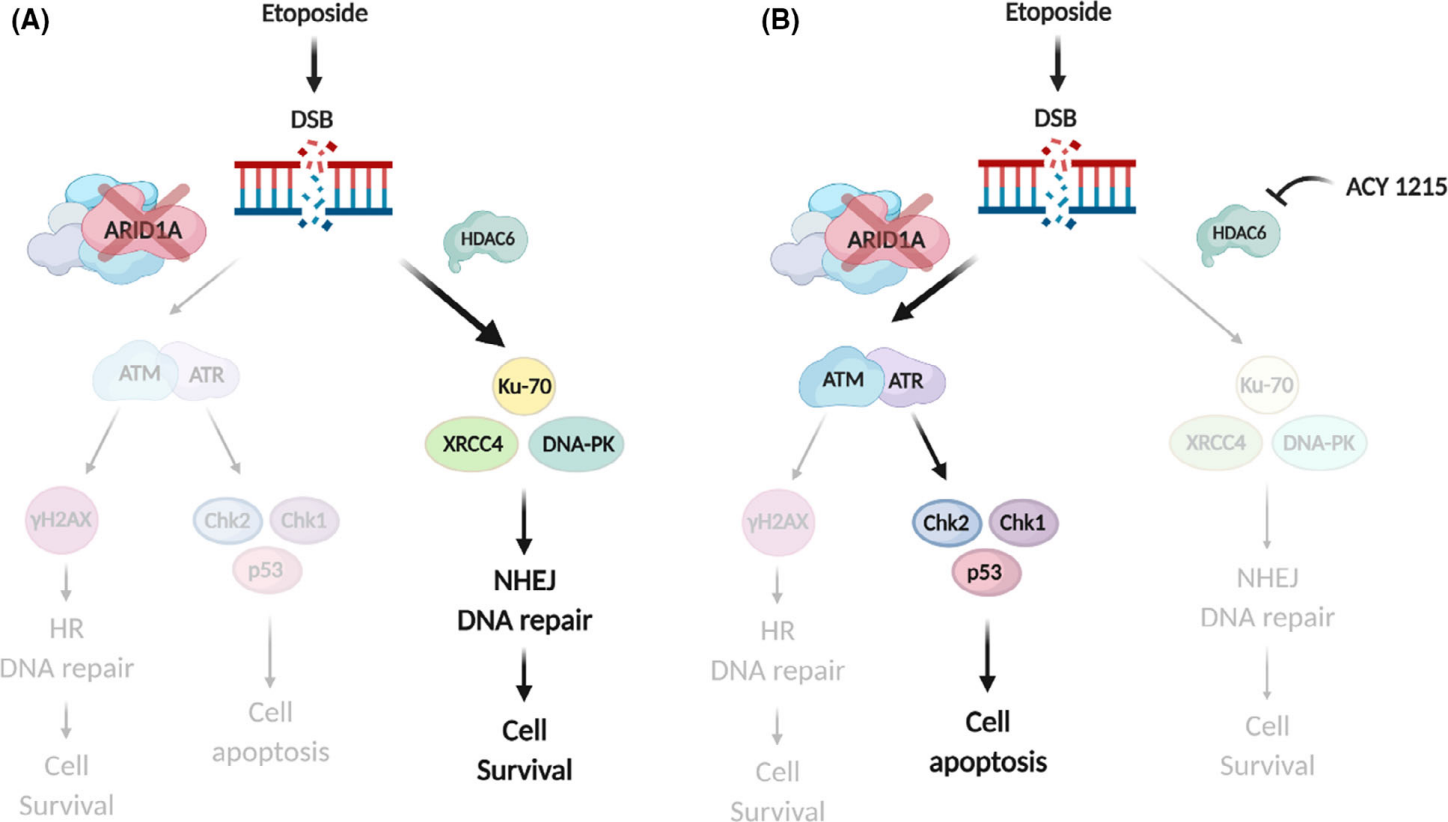
Schematic model proposed. ARID1A deficiency causes cells rebalance or compensatory DNA repair pathways involving HDAC6 for repairing etoposide DSBs DNA breaks. Schematic model of the DNA repair pathways in (A) ARID1A‐depleted cells following etoposide treatment and (B) ARID‐1A‐deficient cells with etoposide and HDAC6 inhibitor treatment. Created with BioRender.com.

Mammalian cells exploit two major DSB repair pathways, the ATM‐dependent HR and the DNA‐PKcs‐mediated NHEJ pathways, each of which harness a unique set of molecular players. The balance between both pathways is essential for genome stability, and disturbances of this equilibrium might lead to carcinogenesis [48]. SWI/SNF chromatin remodelling complex has been reported to participate in the early phase of DSB repair through rapid localization of the DSB site, clearing local nucleosome occupancy, and physically facilitating the recruitment of DNA repair enzymes and other modulators [57]. As shown in the proposed model (Fig. 7), our results indicate that ARID1A is necessary for an early activation of the G2/M checkpoint, after being exposed to DSB‐inducing agents such as etoposide. Thus, depending on the magnitude of the damage, the cells initiate the HR DNA damage repair pathway or undergo apoptosis, if DNA damage cannot be repaired. However, in ARID1A‐defective cells, the NHEJ pathway, in which HDAC6 plays a central role, is a backup pathway for DSB damage repair.

In consonance with other cellular models [44, 51, 58, 59, 60], here we show that ARID1A deficiency upregulates HDAC6 expression, which directly deacetylate the apoptosis‐promoting p53 Lys373/382 acetylation, thus contributing to the apoptotic response blockade and stimulating activation of NHEJ DSB repair pathway.

Since HDAC6 overexpression in *ARID1A*‐mutated cancers has been associated with several chemoresistant factors [59], we wondered whether inhibition of HDAC6 may be a promising therapy for those cancers. Based on this hypothesis, we assessed whether or not the inhibition of HDAC6 expression in ARID1A deficient cells could re‐balance DNA damage response to etoposide treatment, inducing cell death (Fig. 7). The results show that activation of NHEJ pathway, after DSB damage, is dependent on HDAC6 activity in ARID1A deficient cells. Therefore, HDAC6 inhibition selectively promoted apoptosis of ARID1A‐defective cells treated with etoposide. Hence, in this scenario, DNA‐damaging agents that induce similar types of DNA breaks should exhibit synergy with HDAC6 inhibitors in *ARID1A*‐mutated tumours, including endometrial cancer.

Furthermore, the data provided demonstrate an important role for ARID1A in the preservation of endometrial epithelial cell identity and EMT regulation. Partial or complete EMT has been associated with invasive phenotype in endometrial cancer [36], since EMT process allows cells to move away from their epithelial cell community and to integrate into the surrounding tissue, even at remote locations. The results presented here show that ARID1A‐defective cells display a decrease of epithelial protein markers and an increase of mesenchymal proteins. Moreover, these changes endow ARID1‐deficient cells with migratory and invasion capacities, contributing to an acquisition of malignant phenotype and to a metastatic spread. However, when cells expressing ARID1A knockdown levels are treated with the HDAC6 inhibitor ACY1215, ARID1A‐deficient cells undergo a reversed EMT phenotype, by re‐expressing epithelial markers and diminishing migratory and invasion capabilities. Furthermore, *in vivo*, inhibition of HDAC6 expression in ARID1A mutant cells drastically reduces cellular dissemination. Therefore, the data observed suggest that HDAC6 modulates endometrial epithelial cell identity by regulating the activity of genes involved in transdifferentiating epithelial cells into mesenchymal. In this manner, the mesenchymal phenotype may be maintained during cell colonization of distant sites, where these cells will home and generate a metastasis. In other words, ARID1A through HDAC6 expression regulation, may promote endometrial plasticity by limiting the differentiation capacity of epithelial cells and thus preventing metastasis. As a result, the inhibition of HDAC6 activity represents an exciting therapeutic possibility in high‐grade/stage, invasive or metastatic EC harbouring *ARID1A* mutations, since metastasis is the main cause of death in patients with cancer.

## 5 Conclusions

In summary, our observation demonstrates that ARID1A deficiency accelerates cell cycle transition, cell malignant transformation and anti‐apoptotic effects due to HDAC6 overexpression. Consequently, the use of HDAC6 inhibitors may represent a valuable therapeutic strategy. Noticeably, HDAC6 inhibitors such as ACY1215 are well tolerated and present minimal toxicity in clinical trials [61, 62]. Therefore, our study provides a scientific rationale for potential clinical translation of these findings, purposing clinically applicable HDAC6 inhibitors for *ARID1A*‐mutated EC diagnosed in advanced stages, for which there are currently no effective therapies.

## Conflict of interest

The authors declare no conflict of interest.

## Author contributions

Study conception and design: M‐LC; EN; GS and M‐GX Acquisition and interpretation of data: M‐LC; EN; M‐GX; SP; M‐MN; NR; A‐VM; UI; RI; MA and L‐ND. Bioinformatics analysis: BN and L‐ND. Optimized conditions for the IHC and histopathology analysis: SM; PM; GS and M‐GX. All authors were involved in the writing of the manuscript and gave the submitted version their final approval.

### Peer review

The peer review history for this article is available at https://publons.com/publon/10.1002/1878‐0261.13193.

## Supporting information


**Fig. S1.** Loss of ARID1A expression does not initiate malignant transformation in the HES cell line nor in the *in vivo Cre:ER^T^; Arid1a^f/f^* mice model.

**Fig. S2.** Loss of ARID1A expression in MFE‐296 and HEC‐1A endometrial cancer cell lines enhances tumour growth and progression by a failure in G2/M DNA damage checkpoint.

**Fig. S3.** ARID1A down‐expression promotes EMT process in HEC‐1A endometrial cancer cell line.

**Fig. S4.** ARID1A deficiency omits DSB DNA damage apoptotic response induced by etoposide in HEC‐1 cell line.

**Fig. S5.** Inhibition of HDAC6 expression suppress migratory and invasive capacities of HEC‐1A and MFE‐296 endometrial cancer cell lines.

**Fig. S6.** Resistance to DSB‐induced apoptosis upon etoposide treatment expression is reversed by ACY1215 treatment in HEC‐1A cells.

**Fig. S7.** Quantification of western blot plots.

Supplementary Material

## Data Availability

The data that support the findings of this study are available on request from the corresponding author.
