# Supplementary figures and images for: ARID1A‐deficient cells require HDAC6 for progression of endometrial carcinoma
Source: Mol Oncol. 2022 Mar 2;16(11):2235–59. doi: 10.1002/1878-0261.13193 (PMC9168762; doi:10.1002/1878-0261.13193)

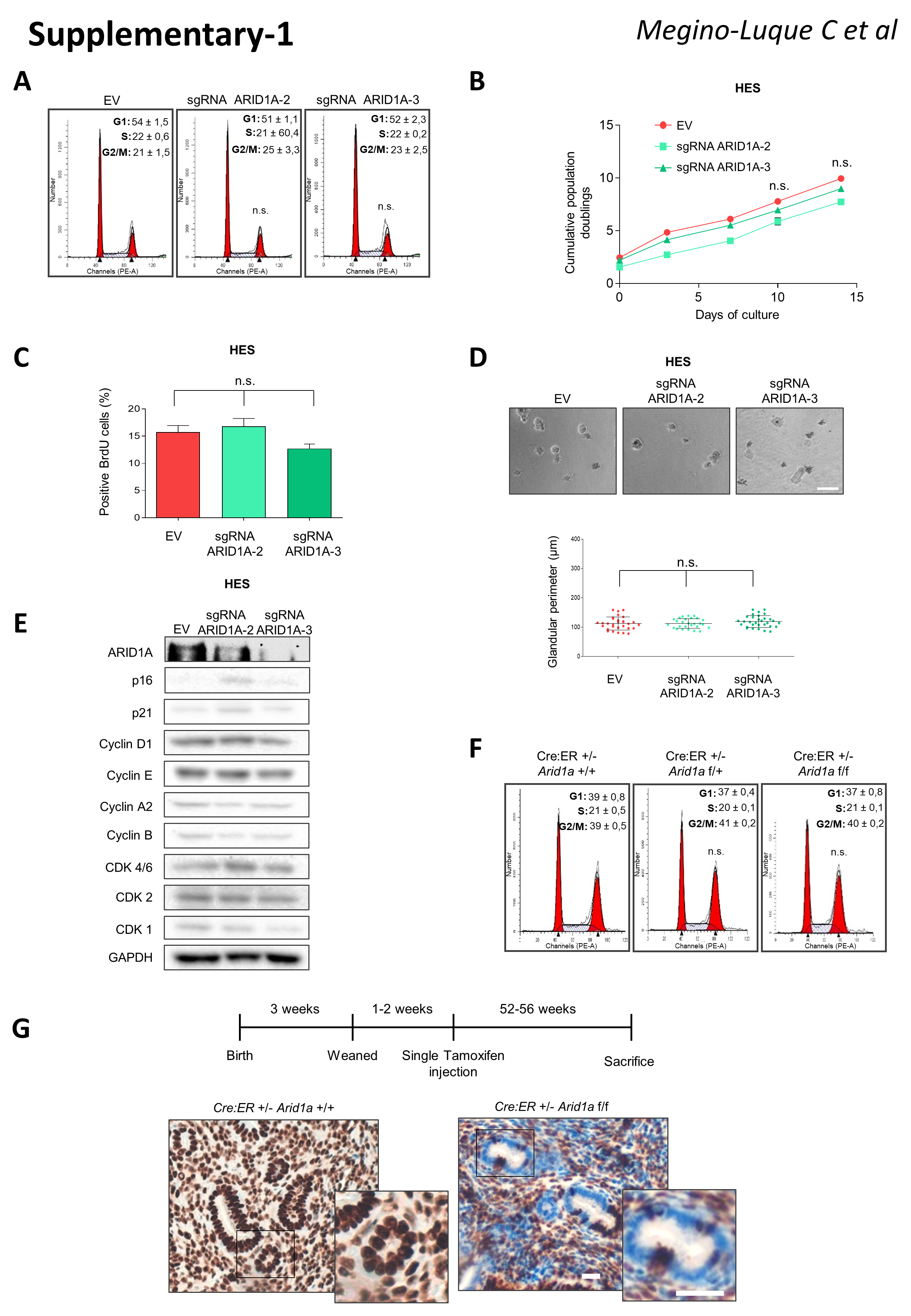

Supplement: Supplementary file 1 — Fig. S1. Loss of ARID1A expression does not initiate malignant transformation in the HES cell line nor in the in vivo Cre:ERT; Arid1af/f mice model. [file MOL2-16-2235-s002.png]

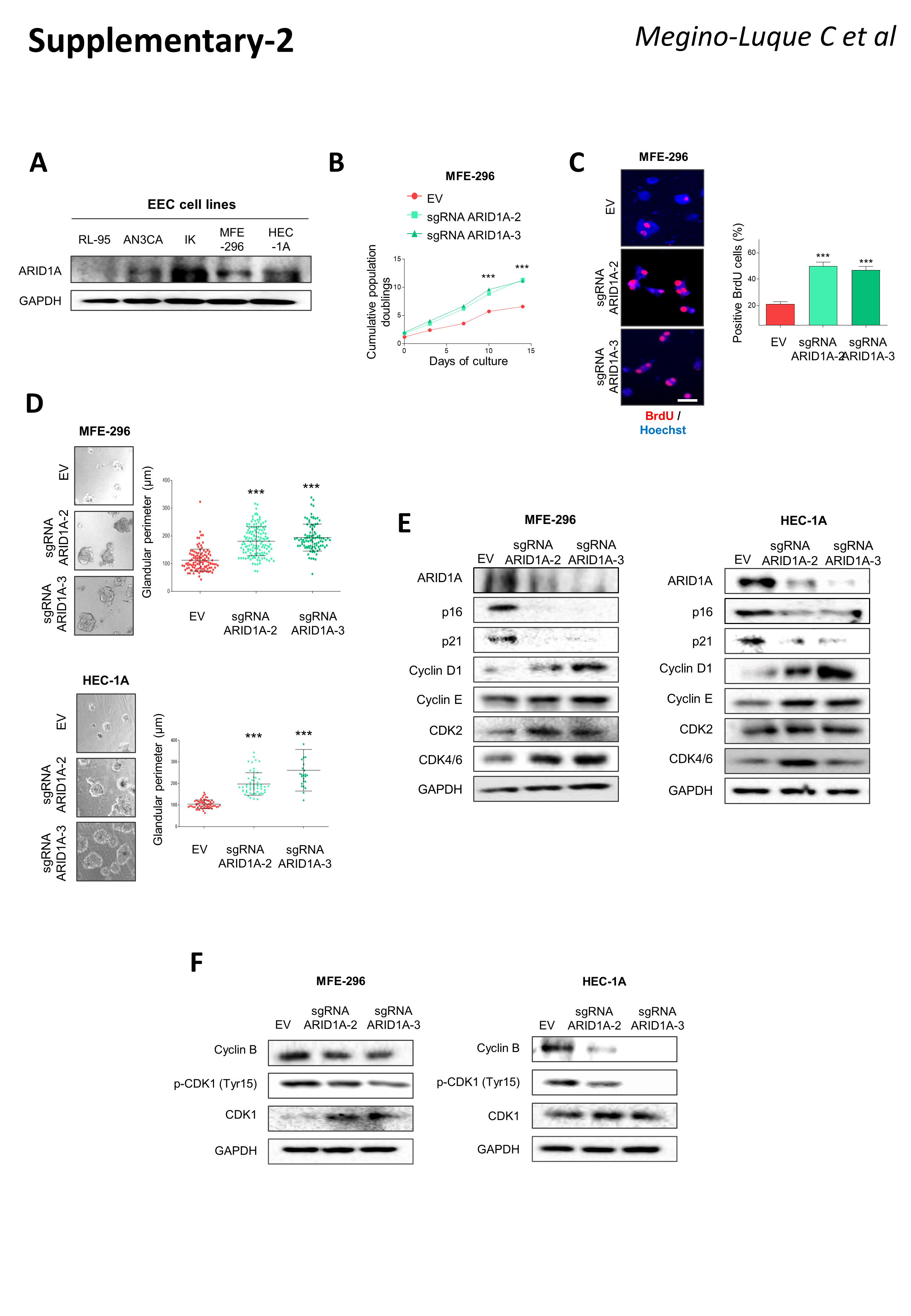

Supplement: Supplementary file 2 — Fig. S2. Loss of ARID1A expression in MFE‐296 and HEC‐1A endometrial cancer cell lines enhances tumour growth and progression by a failure in G2/M DNA damage checkpoint. [file MOL2-16-2235-s001.png]

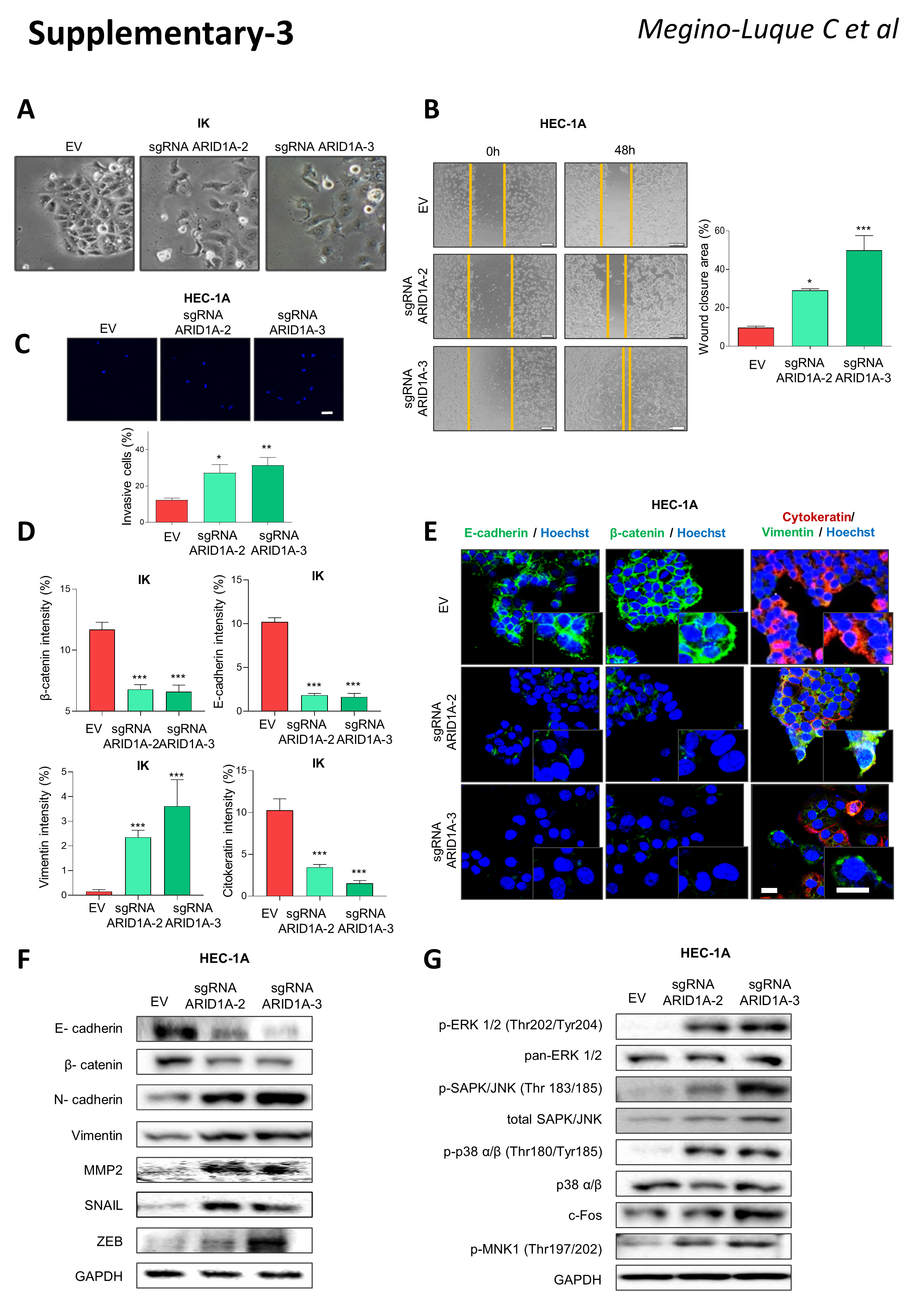

Supplement: Supplementary file 3 — Fig. S3. ARID1A down‐expression promotes EMT process in HEC‐1A endometrial cancer cell line. [file MOL2-16-2235-s003.png]

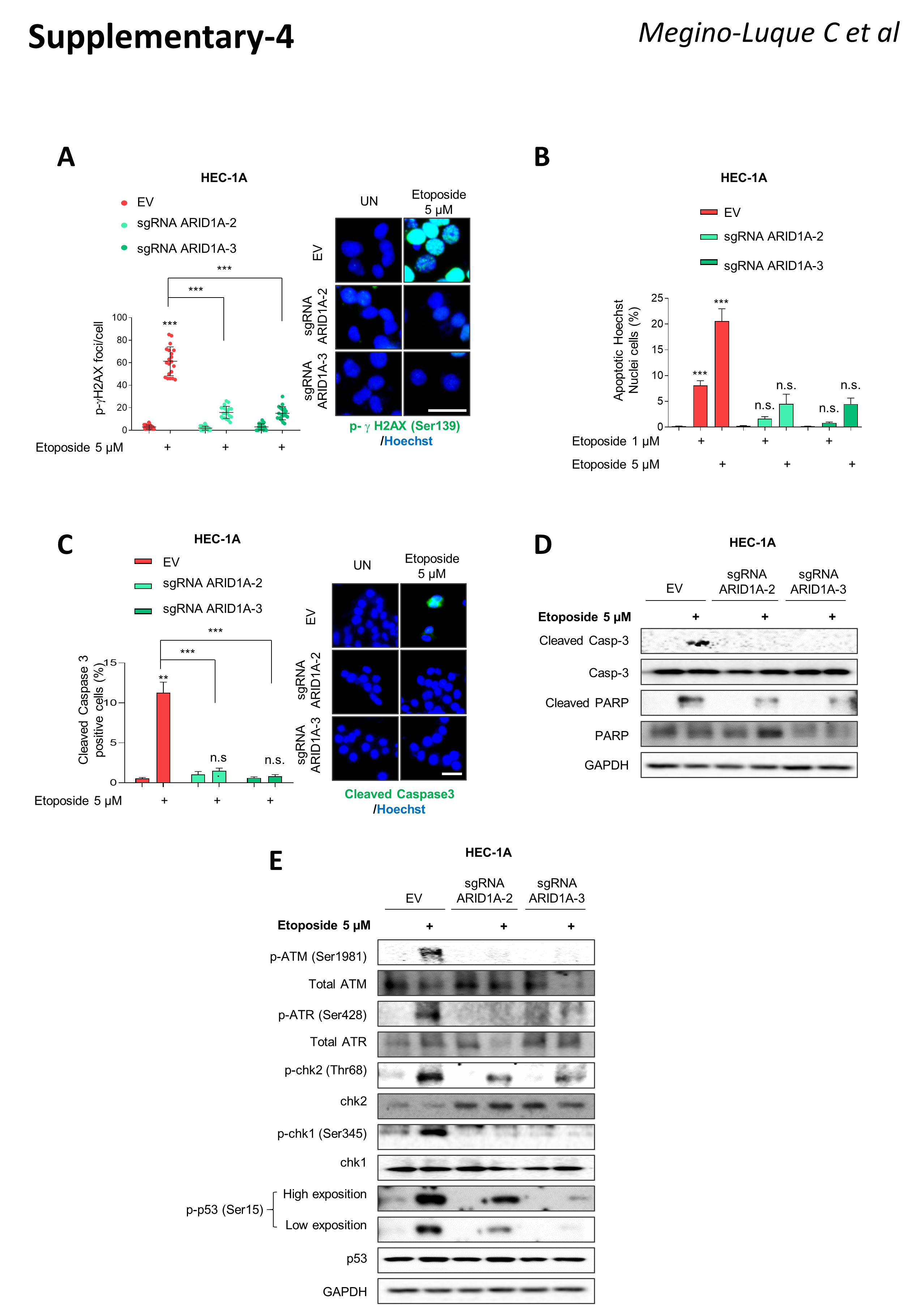

Supplement: Supplementary file 4 — Fig. S4. ARID1A deficiency omits DSB DNA damage apoptotic response induced by etoposide in HEC‐1 cell line. [file MOL2-16-2235-s005.png]

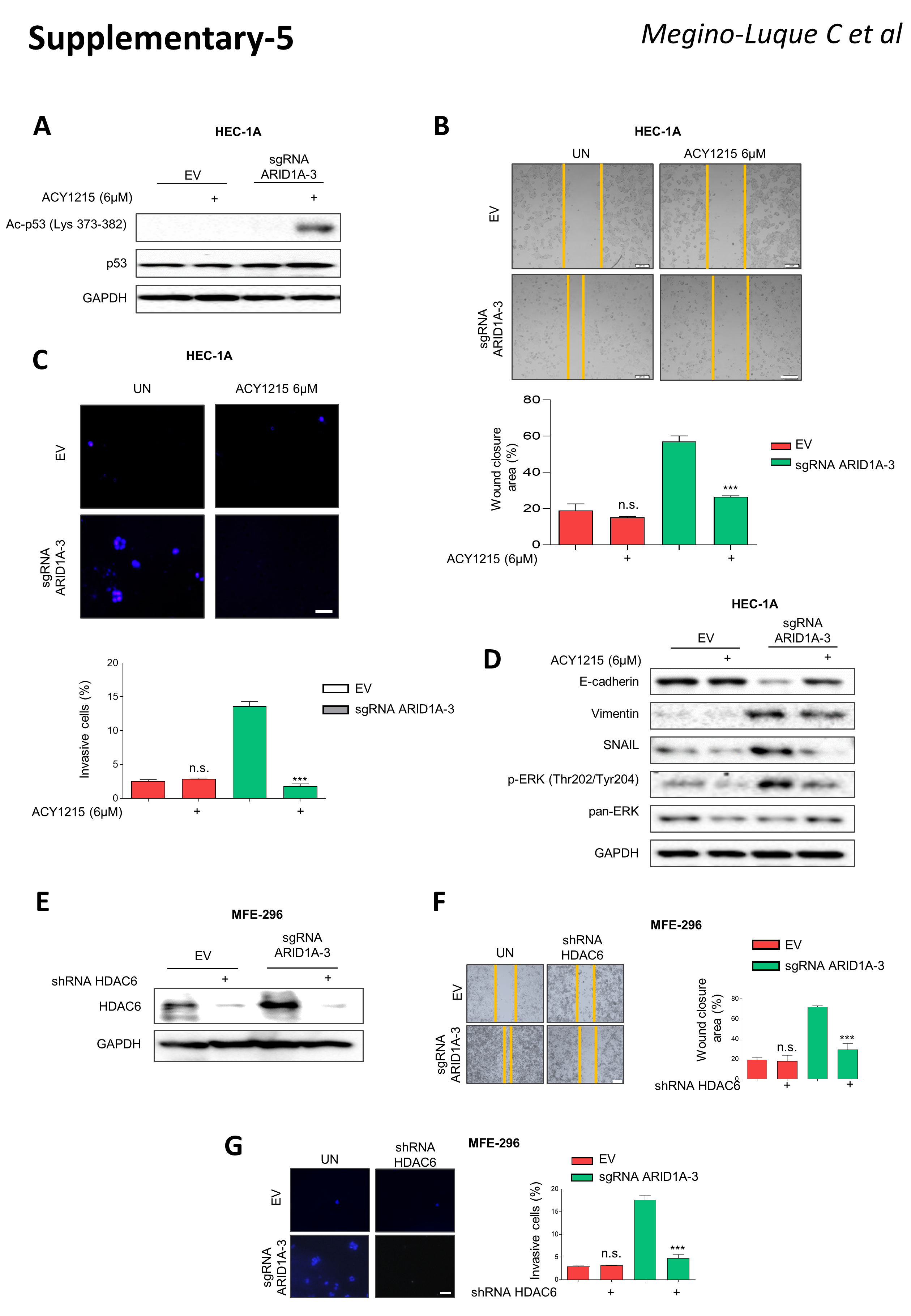

Supplement: Supplementary file 5 — Fig. S5. Inhibition of HDAC6 expression suppress migratory and invasive capacities of HEC‐1A and MFE‐296 endometrial cancer cell lines. [file MOL2-16-2235-s008.png]

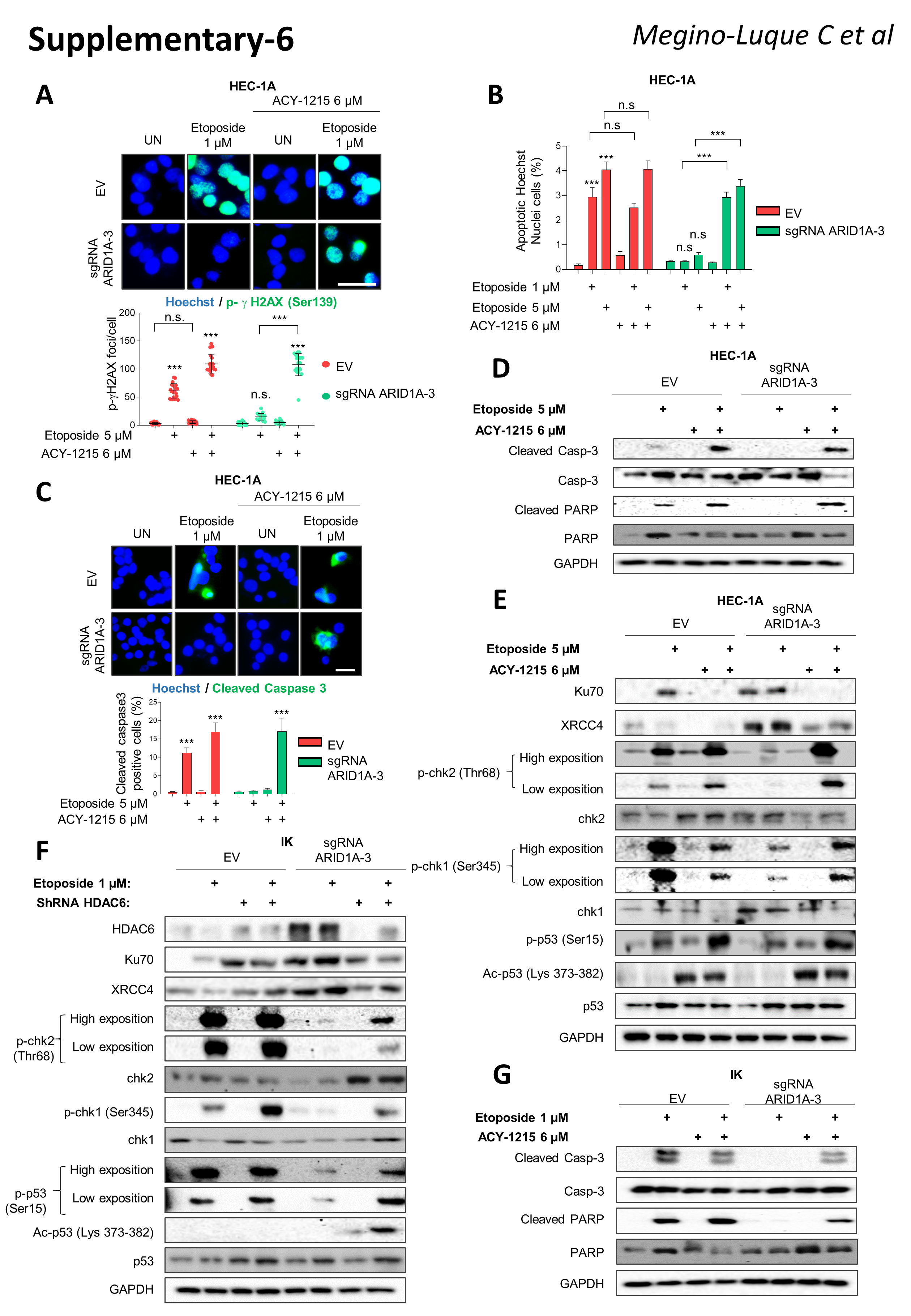

Supplement: Supplementary file 6 — Fig. S6. Resistance to DSB‐induced apoptosis upon etoposide treatment expression is reversed by ACY1215 treatment in HEC‐1A cells. [file MOL2-16-2235-s006.png]

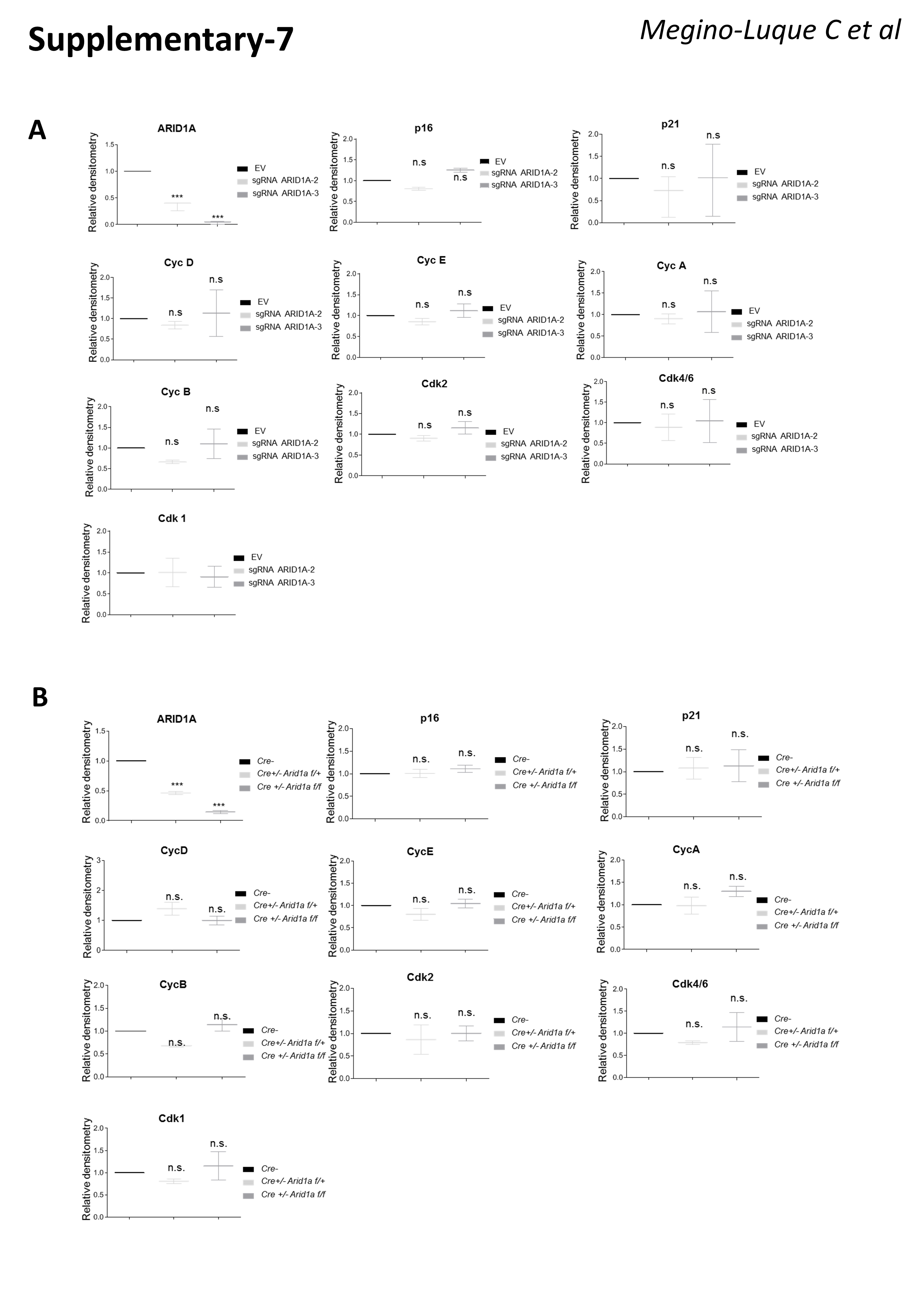

Supplement: Supplementary file 7 — Fig. S7. Quantification of western blot plots. [file MOL2-16-2235-s004.png]
