# Supplementary material for: ARID1A‐deficient cells require HDAC6 for progression of endometrial carcinoma
Source: Mol Oncol. 2022 Mar 2;16(11):2235–59. doi: 10.1002/1878-0261.13193 (PMC9168762; doi:10.1002/1878-0261.13193)
Supplement: Supplementary file 8 — Supplementary Material [file MOL2-16-2235-s007.docx]

***ARID1A*-deficient cells require HDAC6 for progression of endometrial carcinoma**

**Supplementary Figures legends**

**Figure supplementary 1: Loss of ARID1A expression does not initiate malignant transformation in the HES cell line nor in the *in vivo* *Cre:ER^T^; Arid1a^f/f^* mice model.** **A)** Representative histograms and quantification of cell cycle distribution of mice endometrial epithelial cells infected with lentiviruses carrying sgRNA against Arid1a (lentiCRISPRv2-ARID1A.2 and lentiCRISPRv2-ARID1A.3). **B)** Cumulative population doubling levels of HES cells infected with lentiviruses carrying sgRNA against ARID1A (lentiCRISPRv2-ARID1A.2 and lentiCRISPRv2-ARID1A.3) after 15 days of culture. **C)** Quantification of BrdU-positive HES cells infected with lentiviruses carrying sgRNA against ARID1A (lentiCRISPRv2-ARID1A.2 and lentiCRISPRv2-ARID1A.3). **D)** Representative phase contrast images and measurement of gland perimeter corresponding to 3D cultures of HES cells infected with lentiviruses carrying sgRNA against ARID1A (lentiCRISPRv2-ARID1A.2 and lentiCRISPRv2-ARID1A.3). Scale bars: 1000 μm. **E)** Representative images of western blot analysis of HES cells infected with lentiviruses carrying sgRNA against ARID1A (lentiCRISPRv2-ARID1A.2 and lentiCRISPRv2-ARID1A.3) showing ARID1A, p16, p21, Cyclin D1, Cyclin E, Cyclin A2, Cyclin B, CDK2, CDK4/6 and CDK1 protein expression. GAPDH was used as loading control. **F)** Representative histograms and quantification of cell cycle distribution of *Cre:ER^T^; Arid1a^+/+^, Cre:ER^T^; Arid1a^f/+^ or Cre:ER^T^; Arid1a^f/f^* epithelial endometrial cells.**G)** Above, schematic diagram showing the *in vivo* experimental workflow. In brief, mice were weaned 3 weeks after birth and after 5-7 weeks of weaning were injected with a single dose of tamoxifen to achieve Arid1a ablation. Mice were sacrificed 52-56 weeks later. Below, representative images showing ARID1A immunostaining performed on serial endometrial tissue sections from *Cre:ER^T^; Arid1a^+/+^, or Cre:ER^T^; Arid1a ^f/f^* mice. Scale bars: 100 μm. Graph values are the mean and error bars represented as mean ± S.E.M. Statistical analysis was performed using one-way ANOVA analysis followed by Tukey’s multiple comparison test. n.s. (not significant p≥0,05). Results shown are representative of three independent experiments with three technical replicates per experiment. E.V.: Empty vector.

**Figure supplementary 2:** **Loss of ARID1A expression in MFE-296 and HEC-1A endometrial cancer cell lines enhances tumour growth and progression by a failure in G2/M DNA damage checkpoint. A)** Representative images of western blot analysis of ARID1A levels of RL-95, IK, AN3CA, HEC-1A and MFE-296 EEC cell lines. GAPDH was used as a loading control. **B)** Cumulative population doubling levels of MFE-296 cells infected with lentiviruses carrying sgRNA against *ARID1A* (lentiCRISPRv2-ARID1A.2 and lentiCRISPRv2-ARID1A.3) after 15 days of culture. **C)** Representative images and quantification of BrdU-positive MFE-296 cells infected with lentiviruses carrying sgRNA against *ARID1A* (lentiCRISPRv2-ARID1A.2 and lentiCRISPRv2-ARID1A.3). Scale bars: 25 μm. **D)** Representative phase contrast images and measurement of gland perimeter corresponding to 3D cultures of MFE-296 and HEC-1A cells infected with lentiviruses carrying sgRNA against *ARID1A* (lentiCRISPRv2-ARID1A.2 and lentiCRISPRv2-ARID1A.3). Scale bars: 100 μm. **E)** Representative images of western blot analysis of MFE-296 and HEC-1A cells infected with lentiviruses carrying sgRNA against *ARID1A* (lentiCRISPRv2-ARID1A.2 and lentiCRISPRv2-ARID1A.3) showing ARID1A, p16, p21, Cyclin D1, Cyclin E, Cyclin A2, CDK2 and CDK4/6 protein expression. GAPDH was used as a loading control. **F)** Representative images of western blot analysis of MFE-296 and HEC-1A cells infected with lentiviruses carrying sgRNA against *ARID1A* (lentiCRISPRv2-ARID1A.2 and lentiCRISPRv2-ARID1A.3) showing Cyclin B, p-CDK1 (Tyr15) and total CDK1. GAPDH was used as loading control. Graph values are the mean and error bars represented as mean ± S.E.M. Statistical analysis was performed using one-way ANOVA analysis followed by Tukey’s multiple comparison. **p* < 0.05; ****p* < 0.001. Results shown are representative of three independent experiments with three technical replicates per experiment. E.V.: Empty vector.

**Figure supplementary 3: ARID1A down-expression promotes EMT process in HEC-1A endometrial cancer cell line.** **A)** Representative phase contrast images of live cell morphologies of IK cells infected with lentiviruses carrying sgRNA against *ARID1A* (lentiCRISPRv2-ARID1A.2 and lentiCRISPRv2-ARID1A.3). Scale bars: 10 μm **B)** Representative images at time 0 and 48 h after scratch, of wound-healing assay performed in HEC-1A cells infected with lentiviruses carrying sgRNA against *ARID1A* (lentiCRISPRv2-ARID1A.2 and lentiCRISPRv2-ARID1A.3) (left panel) and quantification of wound closure area between the indicated time (right panel). Scale bars: 200 μm. **C)** Representative images of nuclear Hoechst staining of transwell invasion assay after the cotton swab in HEC-1A cells infected with lentiviruses carrying sgRNA against *ARID1A* (lentiCRISPRv2-ARID1A.2 and lentiCRISPRv2-ARID1A.3) (upper panel) and quantification of Matrigel® invasive cells (bottom plot). Scale bars: 50 μm. **D)** Quantification of E-cadherin, β-catenin, Cytokeratin and Vimentin immunofluorescence intensities in IK cells infected or not with lentiviruses carrying sgRNA against *ARID1A* **E)** Representative images of immunofluorescence against E-cadherin, β-catenin, Cytokeratin, Vimentin, Phalloidin, GM130 and Hoechst in HEC-1A cells infected with lentiviruses carrying sgRNA against *ARID1A* (lentiCRISPRv2-ARID1A.2 and lentiCRISPRv2-ARID1A.3). Magnification images of framed regions of the samples are shown. Scale bars: 25 μm. **F)** Representative immunoblots showing E-cadherin, β-catenin, N-cadherin, Vimentin, MMP2, SNAIL and ZEB protein expression in HEC-1A cells infected with lentiviruses carrying sgRNA against *ARID1A* (lentiCRISPRv2-ARID1A.2 and lentiCRISPRv2-ARID1A.3). GAPDH was used as a loading control. **G)** Western blot analysis of phosphorylated and total ERK 1/2, SAPK/JNK and p38 α/β and c-fos in HEC-1A cells infected with lentiviruses carrying sgRNA against *ARID1A* (lentiCRISPRv2-ARID1A.2 and lentiCRISPRv2-ARID1A.3). GAPDH was used as loading control. Graph values are the mean and error bars represented as mean ± S.E.M. Statistical analysis was performed using one-way ANOVA analysis followed by the Tukey’s multiple comparison. **p* < 0.05; ****p* < 0.001. Results shown are representative of three independent experiments with three technical replicates per experiment. E.V.: Empty vector.

**Figure supplementary 4:** **ARID1A deficiency omits DSB DNA damage apoptotic response induced by etoposide in HEC-1 cell line. A)** HEC-1A cells infected with lentiviruses carrying sgRNA against *ARID1A* (lentiCRISPRv2-ARID1A.2 and lentiCRISPRv2-ARID1A.3) treated or not with Etoposide 5 µM during 36h. Right panel shows representative images of cell cultures immunofluorescence against p-Histone γH2AX and Hoechst to show DNA damage in cells under the indicated conditions. Left plot indicates quantification of positive staining for p-γH2AX foci/cell in cells exposed to the described treatments. Scale bars: 25 μm. **B)** Quantification of cells displaying apoptotic nuclei morphologies exposed by Hoechst staining under the indicated conditions. **C)** Representative images and quantification of immunostaining for Cleaved Caspase-3 in HEC-1A cells infected with lentiviruses carrying sgRNA against *ARID1A* (lentiCRISPRv2-ARID1A.2 and lentiCRISPRv2-ARID1A.3) and control cells, untreated or treated with Etoposide 5 µM during 36h. Nuclei were evidenced by Hoechst staining. Scale bars: 25 μm. **D)** Representative immunoblots showing cleaved and total Caspase-3 and PARP protein expression in HEC-1A cells infected with lentiviruses carrying sgRNA against *ARID1A* (lentiCRISPRv2-ARID1A.2 and lentiCRISPRv2-ARID1A.3) and control HEC-1A cells exposed to the described treatments. GAPDH was used as a loading control. **E)** Representative immunoblots showing phosphorylated ATM (Ser1981), ATR (Ser428) chk2 (Thr68), chk1 (Ser345) and p53 (Ser15), and their respective total protein levels in HEC-1A cells infected with lentiviruses carrying sgRNA against *ARID1A* (lentiCRISPRv2-ARID1A.2 and lentiCRISPRv2-ARID1A.3) and control cells after 36h of Etoposide 5 µM exposition. GAPDH was used as a loading control. Graph values are the mean and error bars represented as mean ± S.E.M. Statistical analysis was performed using two-way ANOVA analysis followed Bonferroni post hoc analysis. ***p* < 0.01; ****p* < 0.001, n.s. (not significant p≥0,05). Results shown are representative of three independent experiments with three technical replicates per experiment. E.V.: Empty vector.

**Figure supplementary 5: Inhibition of HDAC6 expression suppress migratory and invasive capacities of HEC-1A and MFE-296 endometrial cancer cell lines. A)** Representative immunoblotting images of Ac-p53 (Lys 373-382) and total p53 protein expression in HEC-1A cells infected with lentiviruses carrying sgRNA against *ARID1A* (lentiCRISPRv2-ARID1A.3) and control cells, untreated or treated with ACY-1215 6 µM during 48 hours. GAPDH was used as a loading control. **B)** Representative images at time 0 and 48 hours after scratch (top) of wound-healing assay performed in HEC-1A cells infected with lentiviruses carrying sgRNA against *ARID1A* (lentiCRISPRv2-ARID1A.2 and lentiCRISPRv2-ARID1A.3) and control cells, untreated or treated with ACY-1215 6 µM during 48 hours. Bottom, graph shows quantification of wound closure area between the indicated time. Scale bars: 200 μm. **C)** Representative images of nuclear Hoechst staining of transwell invasion assay after the cotton swab in HEC-1A cells infected with lentiviruses carrying sgRNA against *ARID1A* (lentiCRISPRv2-ARID1A.2 and lentiCRISPRv2-ARID1A.3) and control cells untreated or treated with ACY-1215 6 µM during 48 hours (upper panel), and quantification of Matrigel® invasive cells (bottom plot). Scale bars: 50 μm. **D)** Western blot analysis of E-cadherin, Vimentin, SNAIL, phosphor-ERK (Thr202/Tyr204) and total pan-ERK protein expression in HEC-1A cells infected with lentiviruses carrying sgRNA against *ARID1A* (lentiCRISPRv2-ARID1A.2 and lentiCRISPRv2-ARID1A.3) and control cells untreated or treated with ACY-1215 6 µM during 48 hours. **E)** Western blot analysis of HDAC6 expression in MFE-296 ARID1A deficient (or not) cells transduced with lentiviral particles containing shRNA-scrambled or shRNA-HDAC6. GAPDH was used as a loading control. **F)** Representative images at time 0 and 48 h after scratch (left) of wound-healing assay performed in MFE-296 ARID1A deficient (or not) cells transduced with lentiviral particles containing shRNA-scrambled or shRNA-HDAC6. Right, graph shows quantification of wound closure area between the indicated conditions and time. Scale bars: 200 μm. **G**) Representative images of nuclear Hoechst staining of transwell invasion assay after the cotton swab in MFE-296 ARID1A deficient (or not) cells transduced with lentiviral particles containing shRNA-scrambled or shRNA-HDAC6 (left panel), and quantification of Matrigel® invasive cells (right plot). Scale bars: 50 μm. Graph values are the mean and error bars represented as mean ± S.E.M. Statistical analysis was performed using two-way ANOVA analysis followed by Bonferroni post hoc analysis. ****p* < 0.001, n.s. (not significant p≥0,05). Results shown are representative of three independent experiments with three technical replicates per experiment. E.V.: Empty vector.

**Figure supplementary 6: Resistance to DSB-induced apoptosis upon etoposide treatment expression is reversed by ACY1215 treatment in HEC-1A cells. A)** HEC-1A cells infected with lentiviruses carrying sgRNA against *ARID1A* (lentiCRISPRv2-ARID1A.3) treated or not with Etoposide 5 µM, ACY-1215 6µM or combination of both, during 36 hours. Upper panel shows representative images of cell cultures immunofluorescence against p-Histone γH2AX and Hoechst to show DNA damage in cells under the indicated conditions. Bottom plot indicates quantification of positive staining for p-γH2AX foci/cell in cells exposed to the described treatments. Scale bars: 25 μm. **B)** Quantification of cells displaying apoptotic nuclei morphologies exposed by Hoechst staining under the same conditions. **C)** Representative images (upper panel) and quantification (bottom plot) of immunostaining for Cleaved Caspase-3 in HEC-1A cells infected with lentiviruses carrying sgRNA against *ARID1A* (lentiCRISPRv2-ARID1A.3) and control cells untreated or treated with Etoposide 5 µM, ACY-1215 6µM or combination of both, during 36h. Nuclei were evidenced by Hoechst staining. Scale bars: 25 μm. **D)** Western blot analysis of cleaved and total Caspase 3 and PARP protein expression in HEC-1A cells infected with lentiviruses carrying sgRNA against *ARID1A* (lentiCRISPRv2-ARID1A.3) treated or not with Etoposide 5 µM, ACY-1215 6µM or combination of both, during 36h. GAPDH was used as a loading control. **E)** Western blot analysis of Ku70, XRCC4, phosphor chk2 (Thr68), phospho-chk1 (Ser345), phosphor-p53 (Ser15), Acetil-p53 (Lys373-382) and their total protein expressions in HEC-1A cells infected with lentiviruses carrying sgRNA against *ARID1A* (lentiCRISPRv2-ARID1A.3) treated or not with Etoposide 5 µM, ACY-1215 6 µM or combination of both, during 36h. GAPDH was used as a loading control. **F)** Western blot analysis of Ku70, XRCC4, phosphor chk2 (Thr68), phospho-chk1 (Ser345), phosphor-p53 (Ser15), Acetil-p53 (Lys373-382) and their total protein expressions in IK cells infected with lentiviruses carrying sgRNA against *ARID1A* (lentiCRISPRv2-ARID1A.3) transfected with HDAC6 shRNA and treated or not with Etoposide 5 µM, ACY-1215 6 µM or combination of both, during 36h. GAPDH was used as a loading control. **G)** Western blot analysis of cleaved and total Caspase 3 and PARP protein expression in IK cells infected with lentiviruses carrying sgRNA against *ARID1A* (lentiCRISPRv2-ARID1A.3) transfected with HDAC6 shRNA and treated or not with Etoposide 5 µM, ACY-1215 6 µM or combination of both, during 36h. GAPDH was used as a loading control. Graph values are the mean and error bars represented as mean ± S.E.M. Statistical analysis was performed using two-way ANOVA analysis followed Bonferroni post hoc analysis. ****p* < 0.001, n.s. (not significant p≥0,05). Results shown are representative of at least three independent experiments with three technical replicates per experiment. E.V.: Empty vector.

**Figure supplementary 7: Quantification of western blot plots. A)** Representative graphs showing quantification of immunoblotting of ARID1A, p16, p21, Cyclin D, Cyclin E, Cyclin A, Cyclin B, Cdk2, Cdk1 and Cdk4/6 from 3D cultures of mouse endometrial epithelial cells infected with lentiviruses carrying sgRNA against Arid1a (lentiCRISPRv2-ARID1A.2 and lentiCRISPRv2-ARID1A.3). **B)** Representative graphs showing quantification of immunoblotting of ARID1A, p16, p21, CycD, CycE, CycA, CycB, Cdk2, Cdk1 and Cdk4/6 from 3D cultures of Cre: ER^T^; Arid1a+/+ , Cre:ERT; Arid1af/+ or Cre:ERT; Arid1af/f epithelial endometrial cells. **C)** Representative graphs showing quantification of immunoblotting of ARID1A, p16, p21, CycD, CycE, CycA, Cdk2, and Cdk4/6 of IK cells infected with lentiviruses carrying sgRNA against *ARID1A* (lentiCRISPRv2-ARID1A.3). **D)** Representative graphs showing quantification of immunoblotting of ARID1A, CycB, p-Cdk1 (Tyr15), Cdk1, p-ATM (Ser1981), ATM, ATR (Ser428), ATR, p-Chk2 (Thr68), Chk2, p-Chk1 (Ser345), p-p53 (Ser15), Ac-p53 (Lys 373-382), p53, Cdc25C and pH3 (Ser10) of IK cells infected with lentiviruses carrying sgRNA against *ARID1A* (lentiCRISPRv2-ARID1A.3). **E)** Representative graphs showing quantification of immunoblotting of E-cadherin, β-catenin, N-cadherin, Vimentin, SNAIL, MMP2 and ZEB1 of IK cells infected with lentiviruses carrying sgRNA against *ARID1A* (lentiCRISPRv2-ARID1A.3). **F)** Representative graphs showing quantification of immunoblotting of p-ERK (Thr202/Tyr204), pan-ERK, p-SAPK/JNK (Thr 183/185), SAPK/JNK, p-p38 α/β (Thr180/Tyr185), p38 α/β, c-fos and p-MNK1 (Thr197/202) of IK cells infected with lentiviruses carrying sgRNA against *ARID1A* (lentiCRISPRv2-ARID1A.3). **G)** Representative graphs showing quantification of immunoblotting of total and cleaved Casp3 and PARP of IK cells infected with lentiviruses carrying sgRNA against ARID1A (lentiCRISPRv2-ARID1A.2 and lentiCRISPRv2-ARID1A.3) treated or not with Etoposide 5 µM during 36h. **H)** Representative graphs showing quantification of immunoblotting of p-ATM (Ser1981), ATM, ATR (Ser428), ATR, p-Chk2 (Thr68), Chk2, p-Chk1 (Ser345), p-p53 (Ser15) and p53 of IK cells infected with lentiviruses carrying sgRNA against ARID1A (lentiCRISPRv2-ARID1A.2 and lentiCRISPRv2-ARID1A.3) treated or not with Etoposide 5 µM during 36h. **I)** Representative graphs showing quantification of immunoblotting of ARID1A and HDAC6 of IK and HEC-1A cells infected or not with lentiCRISPRv2-ARID1A.3. **J)** Representative graphs showing quantification of immunoblotting of Ac-p53 (Lys 373-382) and p53 of IK cells infected or not with lentiCRISPRv2-ARID1A.3 after ACY-1215 treatment (6 µM) during 48 hours. **K)** Representative graphs showing quantification of immunoblotting of E-cadherin, Vimentin, SNAIL, p-ERK (Thr202/Tyr204) and pan-ERK of IK cells infected or not with lentiCRISPRv2-ARID1A.3 after ACY-1215 treatment (6 µM) during 48 hours. **L)** Representative graphs showing quantification of immunoblotting of Cleaved and total Casp3 and PARP of IK cells infected or not with lentiCRISPRv2-ARID1A.3 treated or not with Etoposide 5 µM, ACY-1215 6 µM or combination of both, during 36h. **M)** Representative graphs showing quantification of immunoblotting of Ku70, XRCC4, p-Chk2 (Thr68), Chk2, p-Chk1 (Ser345), p-p53 (Ser15) and p53 of IK cells infected or not with lentiCRISPRv2-ARID1A.3 treated or not with Etoposide 5 µM, ACY-1215 6 µM or combination of both, during 36h. Graph values are the mean and error bars represented as mean ± S.E.M. Statistical analysis was performed using one-way or two-way ANOVA analysis followed by Tukey’s multiple comparison test. n.s. (not significant p≥0,05). Results shown are representative of at three independent experiments of three technical replicates per experiment. E.V.: Empty vector.
